# Theoretical Study of Molecular Structure and Physicochemical Properties of Novel Factor Xa Inhibitors and Dual Factor Xa and Factor IIa Inhibitors

**DOI:** 10.3390/molecules21020185

**Published:** 2016-02-04

**Authors:** Milan Remko, Anna Remková, Ria Broer

**Affiliations:** 1 Department of Pharmaceutical Chemistry, Faculty of Pharmacy, Comenius University in Bratislava, Odbojárov 10, SK-832 32 Bratislava, Slovakia; 2 Department of Internal Medicine, Faculty of Medicine, Slovak Medical University, Limbová 12, SK-833 03 Bratislava, Slovakia; aremkova@szu.sk; 3 Department of Theoretical Chemistry, Zernike Institute for Advanced Materials, University of Groningen, Nijenborgh 4, 9747 AG Groningen, The Netherlands; R.Broer@rug.nl

**Keywords:** density functional theory (DFT), anticoagulants, conformation, solvent effect, acidity, lipophilicity, absorption

## Abstract

The geometries and energies of factor Xa inhibitors edoxaban, eribaxaban, fidexaban, darexaban, letaxaban, and the dual factor Xa and thrombin inhibitors tanogitran and SAR107375 in both the gas-phase and aqueous solution were studied using the Becke3LYP/6-31++G(d,p) or Grimme’s B97D/6-31++G(d,p) method. The fully optimized conformers of these anticoagulants show a characteristic l-shape structure, and the water had a remarkable effect on the equilibrium geometry. According to the calculated p*K*a values eribaxaban and letaxaban are in neutral undissociated form at pH 7.4, while fidexaban and tanogitran exist as zwitterionic structures. The lipophilicity of the inhibitors studied lies within a large range of log *P* between 1 and 4. The dual inhibitor SAR107375 represents an improvement in structural, physicochemical and pharmacokinetic characteristics over tanogitran. At blood pH, SAR107375 predominantly exists in neutral form. In contrast with tanogitran, it is better absorbed and more lipophilic and active after oral application.

## 1. Introduction

During the last 65 years or so compounds from the vitamin K antagonists group (warfarin and its derivatives) have been the only available oral anticoagulants [1], but their use is complicated owing to wide inter-individual variability in dose requirements and their narrow therapeutic index [2,3]. Extensive pharmaceutical research in this field for the last 20–30 years has resulted in several new orally active anticoagulants with significant advantages compared to current drugs such as warfarin and low molecular weight heparin for the treatment and prevention of thrombotic diseases. The new anticoagulants studied for venous thromboembolism treatment inhibit factor Xa (FXa) or thrombin (FIIa). Numerous direct, selective fXa and/or thrombin inhibitors are at various stages of development [4,5,6]. Synthetic inhibitors such as betrixaban, razaxaban, eribaxaban, fidexaban, darexaban (YM150), letaxaban (TAK-442) are members of a new class of orally available fXa inhibitors. In addition to the abovementioned direct fXa inhibitors, dual thrombin/fXa inhibitors such as tanogitran, RWJ-445167, and SAR107375 have also been developed [7,8,9]. Despite a number of experimental structural information about binding models of novel anticoagulants targeting thrombin, fXa or both, experimental studies concerned with the systematic comparative studies of their physicochemical and pharmacokinetic parameters are scarce, and most of novel drug were discovered serendipitously [10]. In our previous work, direct fXa inhibitors (apixaban, rivaroxaban, otamixaban, betrixaban, razaxaban, and DX-9065a) and direct inhibitors of thrombin (ximelagatran and dabigatran etexilate) were studied theoretically [11,12].

In the work presented here we used density functional calculations for the detailed investigation of the molecular structure of direct fXa inhibitors edoxaban, eribaxaban, fidexaban, darexaban (YM150), letaxaban (TAK-442) and the dual factor Xa and thrombin inhibitors tanogitran and SAR107375. The effect of the solvent on the molecular structure of these drugs was evaluated using the polarizable continuum method. Our interest was also focused on the evaluation of basic physicochemical and pharmacokinetic properties (such as p*K*a, lipophilicity, solubility, absorption, and polar surface area) of these species. Attention has also been given to comparison of theoretical results with published experimental properties of these drugs in the light of present theories of their action. These and previously studied anticoagulant drugs [11,12] have entered early and advanced stages of clinical development. Thus, the investigation of their molecular structures and physicochemical properties may contribute to understand the relationships between structure and activity and biological properties of novel anticoagulants.

## 2. Results and Discussion

### 2.1. DFT Calculations of Molecular Structures

**Figure 1:**
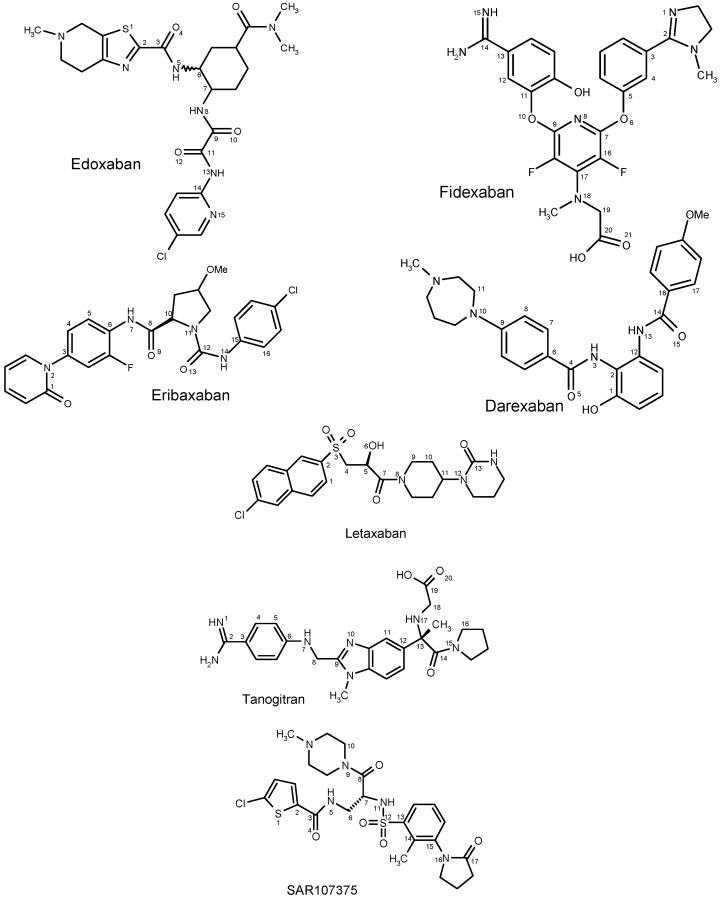
Structure and atom labeling in the anticoagulant drugs studied.

The accurate determination of the geometry of chemical drugs is nowadays accessible through the application of sophisticated quantum chemistry methods [13]. Theoretical chemistry methods are now used in medicinal chemistry and drug design to accurately determine structure and properties of molecules for use in a wide variety of CADD investigations [14,15]. The starting conformations to use in the DFT calculations for these drugs were constructed using the Gauss View program. The spatial orientation of important functional groups of these molecules was defined by selected dihedral angles (α, β, γ, δ, ε, ζ, η, ν, θ, μ, ξ and ρ, Figure 1, Table 1). The important structural parameters (dihedral angles) describing the relative molecular orientation of the studied drugs are presented in the Table 1. The Cartesian coordinates of all inhibitors studied, optimized at the B3LYP level of the density functional theory, are given in Table S1. Subsequent frequency computations of the harmonic vibrational frequencies of the optimized molecules revealed that all the structures obtained were minima (no imaginary frequencies). The equilibrium geometries of the studied drugs computed at the B3LYP and B97D levels of theory are presented in Figure S1. With respect to the nature of drugs studied, their conformational behavior was, in addition to the B3LYP method, also computed using the B97D functional, which includes dispersion. The B97D functional uses the empirical dispersion energy correction specifically designed for an accurate evaluation of van der Waals interactions [16]. Table 2 shows the solvation energies obtained from calculations performed in a vacuum and calculations based on the solvation method. The energy difference between the gas phase and solvated phase was significant for both DFT functionals, B3LYP and B97D, applied in combination with the CPCM solvation model. The largest difference (55.5 kJ/mol) in solvation energies computed with these functionals was found for fidexaban. The different minimum energy conformations computed at the B3LYP and B97D levels of theory for this drug are apparently responsible for this difference (Table 1 and Figure S1).

**Table 1:** Optimized dihedral angles (degrees) of the drugs studied.

Dihedral Angle ^a^	X-ray ^b^	B3LYP/6-31++g(p,d)	B97D/6-31++g(p,d)	B3LYP–CPCM	B97D–CPCM
**(*S*,*R*,*S*)-Edoxaban**					
α[S(1)-C(2)-C(3)-O(4)]		−2.59	−2.77	−0.44	−0.38
β[S(1)-C(2)-C(3)-N(5)]		176.73	176.50	179.30	179.13
γ[C(2)-C(3)-N(5)-C(6)]		173.81	173.90	175.62	175.40
δ[C(3)-N(5)-C(6)-C(7)]		134.17	132.08	122.43	132.05
ε[C(6)-C(7)-N(8)-C(9)]		−147.47	−154.20	−120.05	−144.15
ζ[C(7)-N(8)-C(9)-O(10)]		1.30	2.45	−0.50	3.10
η[C(7)-N(8)-C(9)-C(11)]		−178.53	−177.58	179.45	−177.16
θ[N(8)-C(9)-C(11)]-O(12)]		1.90	2.83	−0.10	1.20
μ[N(8)-C(9)-C(11)]-N(13)]		−178.53	−177.57	179.92	−179.06
ν[C(9)-C(11)]-N(13)-C(14)]		−179.38	−179.90	179.69	−179.71
ξ[C(11)]-N(13)-C(14)-N(15)]		179.16	179.45	−179.78	−179.34
**(*R*,*R*)-Eribaxaban**	2PHB				
α[C(1)-N(2)-C(3)-C(4)]	−82.79	−58.39	−56.15	−96.18	−66.62
β[C(5)-C(6)-N(7)-C(8)]	65.14	4.19	−12.63	5.85	−17.44
γ[C(6)-N(7)-C(8)-O(9)]	−0.09	−4.81	1.63	−2.73	0.77
δ[C(6)-N(7)-C(8)-C(10)]	179.50	176.37	−175.78	178.01	−176.90
ε[N(7)-C(8)-C(10)-N(11)]	1.67	7.42	65.65	4.84	63.15
ζ[C(10)-N(11)-C(12)-O(13)]	−0.39	155.21	164.51	161.65	169.76
η[C(10)-N(11)-C(12)-N(14)]	179.24	−26.05	−16.00	−19.78	−10.78
θ[N(11)-C(12)-N(14)]-C(15)]	168.30	179.48	−178.64	−179.29	−177.55
μ[C(12)-N(14)]-C(15)-C(16)]	−155.47	176.92	174.63	175.98	170.63
H-bond MeO···H-N, Å		2.9338	1.8796	3.0505	1.8373
**Fidexaban**	1FJS				
α[N(1)-C(2)-C(3)-C(4)]	−87.79	−41.41	−39.50	−46.24	−43.11
β[C(4)-C(5)-O(6)-C(7)]	147.64	154.48	161.22	162.20	161.65
γ[C(5)-O(6)-C(7)-N(8)]	115.45	115.13	121.46	115.40	121.26
δ[N(8)-C(9)-O(10)-C(11)]	32.16	−19.64	−106.05	−12.40	−20.05
ε[C(9)-O(10)-C(11)-C(12)]	−120.44	−57.75	9.65	−69.17	−69.45
ζ[C(12)-C(13)-C(14)-N(15)]	−4.70	−21.84	−28.47	−25.67	−25.18
η[C(16)-C(17)-N(18)-C(19)]	−83.09	−132.13	57.97	−133.91	54.12
θ[C(17)-N(18)-C(19)-C(20)]	54.20	85.14	59.75	85.54	80.03
μ[N(18)-C(19)-C(20)-O(21)]	92.47	157.26	−170.74	164.70	162.60
H-bond OH···O, Å		2.1810	2.1949	2.2247	2.2777
H-bond C(=O)OH···N, Å		No interaction	1.5433	No interaction	No interaction
**Darexaban**					
α[C(1)-C(2)-N(3)-C(4)]		42.45	−27.23	44.41	−33.72
β[C(2)-N(3)-C(4)-O(5)]		−3.16	15.93	−5.24	15.21
γ[C(2)-N(3)-C(4)-C(6)]		176.75	−165.63	174.85	−165.63
δ[ N(3)-C(4)-C(6)-C(7)]		0.40	38.24	−1.49	36.32
ε[C(8)-C(9)-N(10)-C(11)]		−15.09	−19.65	−11.84	−15.62
ζ[C(2)-C(12)-N(13)-C(14)]		−55.13	−69.03	−55.92	−67.97
η[C(12)-N(13)-C(14)-O(15)]		6.59	−6.97	5.36	−8.64
θ[C(12)-N(13)-C(14)-C(16)]		−173.43	167.34	−175.12	166.62
μ[N(13)-C(14)-C(16)-C(17)]		156.76	167.28	157.74	164.95
H-bond OH···O, Å		1.6197	1.5871	1.6089	1.5627
H-bond NH···O, Å		1.8333	2.9243	1.8388	3.0555
**(*S*)-Letaxaban**	3KL6				
α[C(1)-C(2)-S(3)-C(4)]	106.69	95.99	99.47	96.34	93.17
β[C(2)-S(3)-C(4)-C(5)]	−76.01	−84.63	−76.11	−77.49	−55.63
γ[S(3)-C(4)-C(5)-O(6)]	71.15	73.11	74.21	72.76	85.44
δ[S(3)-C(4)-C(5)-C(7)]	−170.80	−167.10	−166.10	−168.99	−156.90
ε[C(4)-C(5)-C(7)-N(8)]	177.43	165.01	163.51	144.15	168.58
ζ [C(5)-C(7)-N(8)]-C(9)]	178.57	176.96	171.50	179.43	171.71
η[C(10)-C(11)-N(12)-C(13)]	103.92	124.08	130.11	113.62	110.42
**(*R*)-Tanogitran**					
α[N(1)-C(2)-C(3)-C(4)]		−20.46	−18.73	−24.41	−23.77
β[C(5)-C(6)-N(7)-C(8)]		165.54	−157.23	166.83	−158.63
γ[C(6)-N(7)-C(8)-C(9)]		79.48	49.24	80.05	49.22
δ[N(7)-C(8)-C(9)-N(10)]		−125.14	−115.94	−123.21	−118.50
ε[C(11)-C(12)-C(13)-C(14)]		31.69	38.45	34.23	38.52
ζ[C(11)-C(12)-C(13)-N(17)]		−87.36	−80.82	−83.75	−80.04
η[C(12)-C(13)-N(17)-C(18)]		−177.45	−177.20	−174.71	−174.96
ν[C(13)-N(17)-C(18)-C(19)]		−164.74	−163.43	−164.14	−164.20
θ[N(17)-C(18)-C(19)-O(20)]		16.24	17.08	15.28	16.42
μ[C(12)-C(13)-C(14)-N(15)]		70.94	64.97	72.13	66.74
ξ[C(13)-C(14)-N(15)-C(16)]		−177.86	−171.37	−178.37	−174.10
**(*S*)-SAR107375**					
α[S(1)-C(2)-C(3)-O(4)]		−4.12	−5.85	−2.08	−0.81
β[S(1)-C(2)-C(3)-N(5)]		176.91	175.47	178.21	−179.81
γ[C(2)-C(3)-N(5)-C(6)]		−178.37	−169.31	179.46	−172.09
δ[C(3)-N(5)-C(6)-C(7)]		101.01	90.58	115.31	92.59
ε[N(5)-C(6)-C(7)-C(8)]		−60.05	−57.45	−62.20	−59.89
ζ[C(6)-C(7)-C(8)-N(9)]		−71.35	−78.07	−73.08	−82.25
η[C(7)-C(8)-N(9)-C(10)]		169.79	172.87	173.19	177.96
ν[N(5)-C(6)-C(7)-N(11)]		58.89	63.57	56.60	61.56
θ[C(6)-C(7)-N(11)-S(12)]		106.96	129.39	105.71	135.19
μ[C(7)-N(11)-S(12)-C(13)]		65.20	52.66	62.55	53.58
ξ[N(11)-S(12)-C(13)-C(14)]		62.25	61.92	63.94	66.16
ρ[C(14)-C(15)-N(16)-C(17)]		117.02	141.33	98.19	113.44
σ[N(9)-C(8)-C(7)-N(11)]		168.87	160.95	166.80	156.52
d[O(4)…S(1)], Å		2.9491	2.9876	2.9378	2.9795

^a^ For definition of dihedral angles see Figure 1; ^b^ Protein Data Bank. http://www.rscb.org/pdb/.

**Table 2:** The solvent stability (water) of the drugs studied.

Drug	ΔE^CPCM^, kJ/mol		Gas-phase Dipole Moment, Debye (D)	
	B3LYP–CPCM	B97D–CPCM	B3LYP/6-31++g(p,d)	B97D/6-31++g(p,d)
Edoxaban	−77.48	−73.67	9.54	9.16
Eribaxaban	−75.99	−68.57	1.86	6.61
Fidexaban	−90.60	−37.07	3.33	5.93
Darexaban	−65.66	−72.67	9.89	11.87
Letaxaban	−95.09	−93.43	8.45	7.39
Tanogitran	−101.56	−100.15	5.75	5.31
SAR107375	−89.02	−79.57	6.74	4.62

#### 2.1.1. Edoxaban

**Figure 2:**
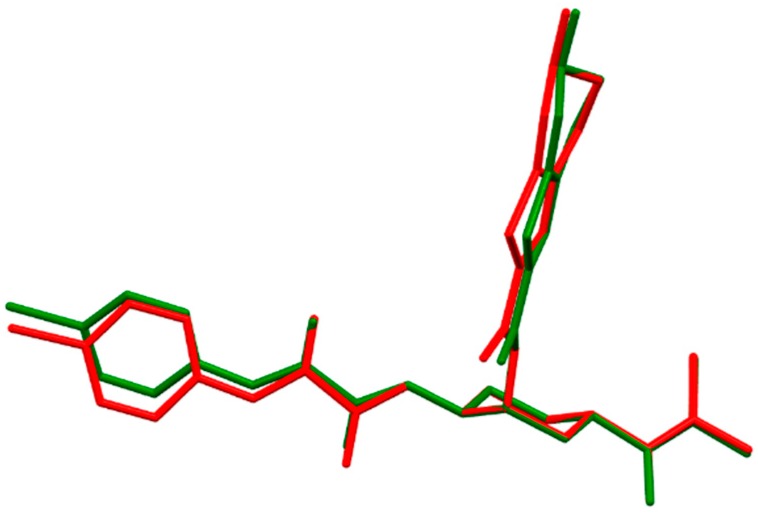
Molecular superimposition of the Becke3LYP optimized molecular structure of edoxaban (green) and hydrated edoxaban (red). For simplicity the hydrogen atoms are omitted.

Edoxaban (*N*-(5-chloropyridin-2-yl)-*N*′-[(1*S*,2*R*,4*S*)-4-[(dimethylamino)carbonyl]-2-[[(5-methyl-4,5,6,7-tetrahydrothiazolo[5,4-c]pyridin-2-yl)carbonyl]amino]cyclohexyl]ethanediamide) is the third fXa inhibitor which was approved by the FDA in 2015 [17]. Edoxaban is a flexible molecule, and its overall space arrangement is defined by 11 dihedral angles (Figure 1). The overall shape of its equilibrium geometry is shown on Figure S1. The calculated molecular conformation of edoxaban is directed by the stereochemistry of the substituents on the central cyclohexane ring. Cyclohexane is present in the more stable chair-shaped conformation [18]. Biologically active edoxaban exists as a pure diastereomer. Substituents at the chiral C-1 and C-4 carbon atoms of cyclohexane adopt the (*S*)*-*configuration and the C-2 substituents of this drug are in the (*R*)-conformation. In this diastereomer, the 3D structure of edoxaban, like other direct fXa inhibitors, is characterized by a unique l-shaped arrangement of the molecule. The chloropyridinyl, amide and *N*,*N*-dimethylcarbamoyl moieties are positioned linearly at the C-1 and C-4 atoms of cyclohexane. The thiazole skeleton is oriented perpendicularly to the plane of the cyclohexane ring to ensure l-shaped 3D structure of this drug (Figure S1). The 3D structure computed with B3LYP and B97D DFT functionals was almost the same with slight differences in the dihedral angles of about 2–4° (Table 1). Upon solvation, the greatest changes by about 10° were observed in the torsion angle ε[C(6)-C(7)-N(8)-C(9)] of the chloropyridinyl moiety. (Figure 2).

#### 2.1.2. Eribaxaban

**Figure 3:**
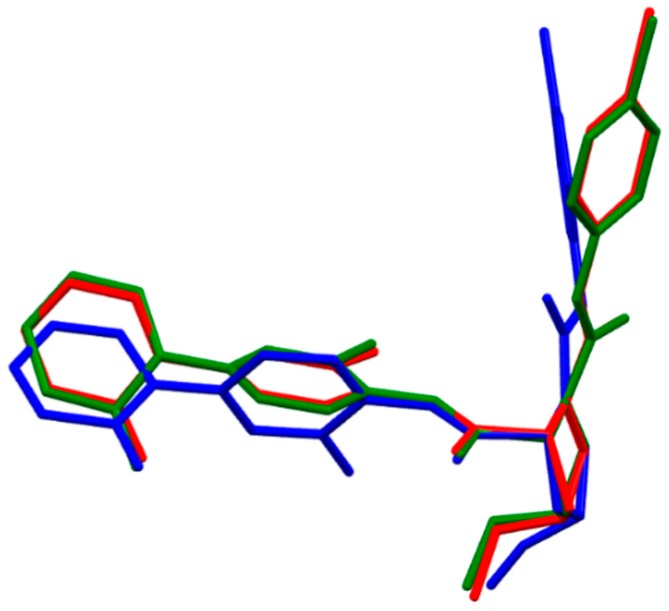
Molecular superimposition of the Becke3LYP optimized molecular structure of (*R,R*)-eribaxaban (green), in solution optimized (*R,R*)-eribaxaban (red) and (*R,R*)-eribaxaban from the co-crystal with coagulation factor Xa, PDB.2PHB (blue). For simplicity the hydrogen atoms are omitted.

(*R,R*)-Eribaxaban ((2R,4R)-N-(4-chlorophenyl)-N-(2-fluoro-4-(2-oxopyridin-1(2H)-yl)phenyl)-4-methoxypyrrolidine-1,2-dicarboxamide] is a proline-based fXa inhibitor discovered by Kohrt *et al.* [19]; it entered phase II clinical trials, although its development was later disrupted in favor of apixaban [20]. The pharmacologically active diastereomer of eribaxaban represents a conformation in which both substituents at the C-2 and C-4 asymmetric carbon atoms of the proline moiety are in in the form of (*R*)-stereoisomers. The full B3LYP optimization of this diastereomer gave the typical l-shaped 3D conformer. A rather different overall shape of this molecule resulted from the B97D calculations. The final energy minimum is fixed via two intramolecular hydrogen bonds formed by the amide moiety of the C-2 proline substituent and the neighboring methoxy and proline amide moieties (Figure S1). Thus, by intramolecular hydrogen bonds, the stabilized conformer is also retained in hydrated eribaxaban (Table 1). The conformation of eribaxaban bound to fXa (PDB file 2PHB) differs from the gas-phase conformation and/or hydrated molecule at two points. The phenylpyridone ring is in the cavity of receptor rotated out of the amide plane by about 65°; the B3LYP calculation predicted coplanarity of this fragment (dihedral angle β[C(5)-C(6)-N(7)-C(8)] = 4.2°). The almost planar arrangement observed for the scaffold containing the chlorophenyl moiety and the proline group in bound eribaxaban is, in the isolated molecule, more relaxed to the periplanar conformation (dihedral angle η[C(10)-N(11)-C(12)-N(14)] = −26°). The structures of the isolated molecule and eribaxaban when bound to fXa (pdb code 2BJ5) are quite different (Figure 3). The large difference in molecular conformations of bound and unbound eribaxaban are also manifested by large energy difference, and the biologically active conformation has a substantially higher energy (640 kJ/mol, B3LYP method) in comparison with the energy of the fully optimized structure. The solvent does not appreciably change the 3D structure of unbound molecule.

#### 2.1.3. Fidexaban

**Figure 4:**
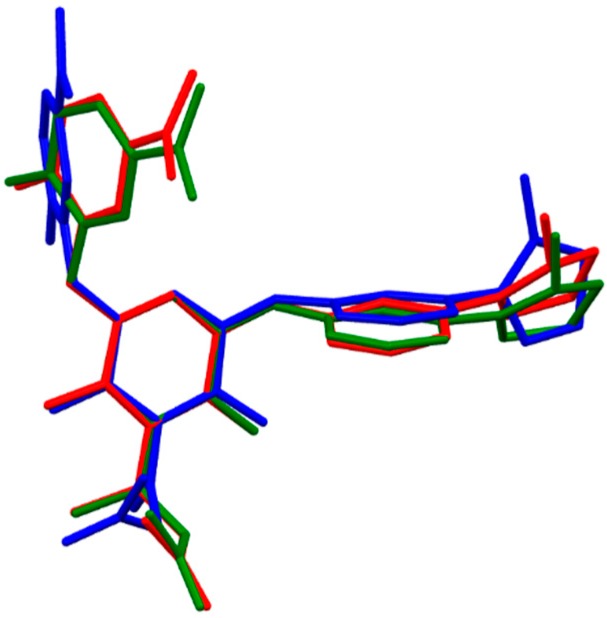
Molecular superimposition of the Becke3LYP optimized molecular structure of fidexaban (green), in solution optimized fidexaban (red) and fidexaban from the co-crystal with coagulation factor Xa, pdb.1FJS (blue). For simplicity the hydrogen atoms are omitted.

Fidexaban (*N*-{2-(5-carbamimidoyl-2-hydroxyphenoxy)-3,5-difluoro-6-[3-(1-methyl-4,5-dihydro-1*H*-imidazol-2-yl)phenoxy]-4-pyridinyl}-*N*-methylglycine) is a parenteral agent that was advanced to human clinical trials [21,22]. The enoxypyridine and phenyoxyimidazoline scaffolds are considered to be in the favored *syn* conformation [21]. The sarcosine at C-4 of the central pyridine ring is maximally extended. The two DFT methods applied describe the molecular structure of fidexaban quite differently (Figure S1). While the skeleton containing the phenoxyimidazoline and pyridine groups was computed by the two methods to have the same general shape (the dihedral angles α[N(1)-C(2)-C(3)-C(4)], β[C(4)-C(5)-O(6)-C(7)] and γ[C(5)-O(6)-C(7)-N(8)] were within 2–6°), the mutual orientation of the phenoxyamidine and sarcosine moieties was completely different. The B3LYP method predicted the most stable conformation in which these moieties are in the maximal extended position, while for the B97D structure, a bent molecule was found (the distance C(=O)O-H···N = 1.54 Å), stabilized by means of intramolecular hydrogen bonds formed by the acidic hydrogen of the sarcosine carboxyl and the basic nitrogen atom of the phenoxyamidine group. The amidine and phenyl groups of the phenoxyamidine moiety form a dihedral angle ζ[C(12)-C(13)-C(14)-N(15)] of about 21° (B3LYP) and 28° (B97D). The structural arrangement around the ether bond connecting the phenoxyamidine and pyridine groups was described completely differently by the B3LYP and B97D methods (the dihedral angle δ[N(8)-C(9)-O(10)-C(11)] of −19.4° (B3LYP) and −106° (B97D); Table 1). These large differences in dihedral angles obtained by two DFT methods could be partially explained by significant overestimation the dispersion in this system. The molecular geometry of hydrated fidexaban treated with the B3LYP functional changed only slightly (Figure 4). However, the dramatic structural rearrangement of fidexaban upon hydration occurred with the B97D functional. The B97D optimized solvated fidexaban resembled the solvated structure of this molecule computed with B3LYP (Table 1). Accordingly, the environmental effect partially compensated overestimated dispersion interaction also manifested in the absence of the intramolecular C(=O)O-H···N interaction in the optimized structure (Table 1, Figure S1). An analysis of crystal structure of the fidexaban-fXa complex (pdf file 1FJS) shows that the phenoxyamidine group accommodates the polar S1 pocket and the hydrophobic part of the drug’s phenoxyimidazoline moiety is located at the hydrophobic S4 site. The final biologically active conformation of fidexaban is governed by a strong salt bridge of amidine group with Asp189 in the S1 pocket [22], which results in a large conformational change to the phenylamidine scaffold of this drug upon complexation with fXa (Figure 4). The corresponding dihedral angles δ[N(8)-C(9)-O(10)-C(11)] and ε[C(9)-O(10)-C(11)-C(12)] are −19.6° and −56.8° for the complexed species and −106° and 9.6° for the isolated molecule, respectively (Table 1). The large conformational differences between conformations of unbound and bound fidexaban could be explained by the intermolecular interactions between fidexaban and receptor. The central pyridine ring represents a rigid scaffold which orients the phenoxyimidazoline moiety towards Trp215 in the S4 pocket, stabilized by an aromatic ring stacking interaction between the fidexaban and the corresponding aromatic amino acid of receptor. The biologically active conformation of fidexaban is less stable by 319 kJ/mol.

#### 2.1.4. Darexaban

Darexaban (*N*-(3-hydroxy-2-{[4-(4-methyl-1,4-diazepan-1-yl)benzoyl]amino}phenyl)-4-methoxybenzamide) is a novel fXa inhibitor without an amidine moiety incorporated into previous fXa inhibitors like fidexaban, otamixaban and betrixaban [23]. Upon oral administration in humans, darexaban is well-absorbed from the gastrointestinal tract, with most of the absorbed drug rapidly metabolized to darexaban glucuronide (M1), the pharmacological activity of which is equipotent to unchanged darexaban [23]. Its development was discontinued in September 2011 [20]. Darexaban contains a central 1,2-phenylenediamide scaffold; the amide groups are for stereochemical reasons twisted out of the phenyl ring plane by about 42° and −55° (B3LYP, dihedral angles α[C(1)-C(2)-N(3)-C(4)] and ζ[C(2)-C(12)-N(13)-C(14)], Table 1), respectively. The 1,4-benzodiazepane moiety is perpendicularly oriented to the phenyl substituent, and together with 4-methoxybenzamide forms a typical U-shaped structure stabilized by means of OH···O=C and NH···O=C intramolecular hydrogen bonds among polar substituents on the central phenyl ring (Table 1, Figure S1). A different 3D structure resulted from the B97D calculations. The terminal 1,4-benzodiazepane and 4-methoxybenzamide groups were more twisted and closer to each other and stabilized via a OH···O=C hydrogen bond (Figure S1). The solvent effect treated within the conductor-like polarizable continuum model (CPCM) did not significantly change the 3D structure of the isolated molecule (Table 1).

#### 2.1.5. Letaxaban

Letaxaban (1-(1-{(2*S*)-3-[(6-chloro-2-naphthyl)sulfonyl]-2-hydroxypropanoyl}-4-piperidinyl)tetrahydro-2(1*H*)-pyrimidinone), also known as TAK-442, is a potent, selective, and orally active factor Xa inhibitor, and is a tetrahydropyrimidin-2(1*H*)-one derivative [24]. Letaxaban was discontinued in May 2011 following disappointing phase II results [20]. The 3D molecular structure computed at the B3LYP level of theory of its biologically active (*S*) enantiomer is shown in Figure S1. Both the B3LYP and G97D functionals predicted almost the same mutual structural arrangement of basic functional groups (Table 1). Letaxaban belongs to the relatively rigid fXa inhibitors, and its minimum energy conformer possesses the typical L-shape required for proper orientation of its terminal functional groups towards the receptor site. Regarding the isolated molecule, the hydrophobic 6-chloronaphthyl group and sulfonyl moiety are in a mutual *anti* arrangement (dihedral angle α[C(1)-C(2)-S(3)-C(4)] is about 96–99°, Table 1), a stable conformation also found in structurally related aromatic sulfonamides [25,26], which orients this part of the drug perpendicularly to the rest of the molecule. The 6-chloronaphthyl group interacts by means of a hydrophobic interaction with the aromatic ring of Tyr228 in the S1 binding site. The 2-hydroxypropanoyl moiety exists in a stable periplanar conformation (the dihedral angles δ[S(3)-C(4)-C(5)-C(7)] and ε[C(4)-C(5)-C(7)-N(8)] are about −167° and 165°, respectively).

**Figure 5:**
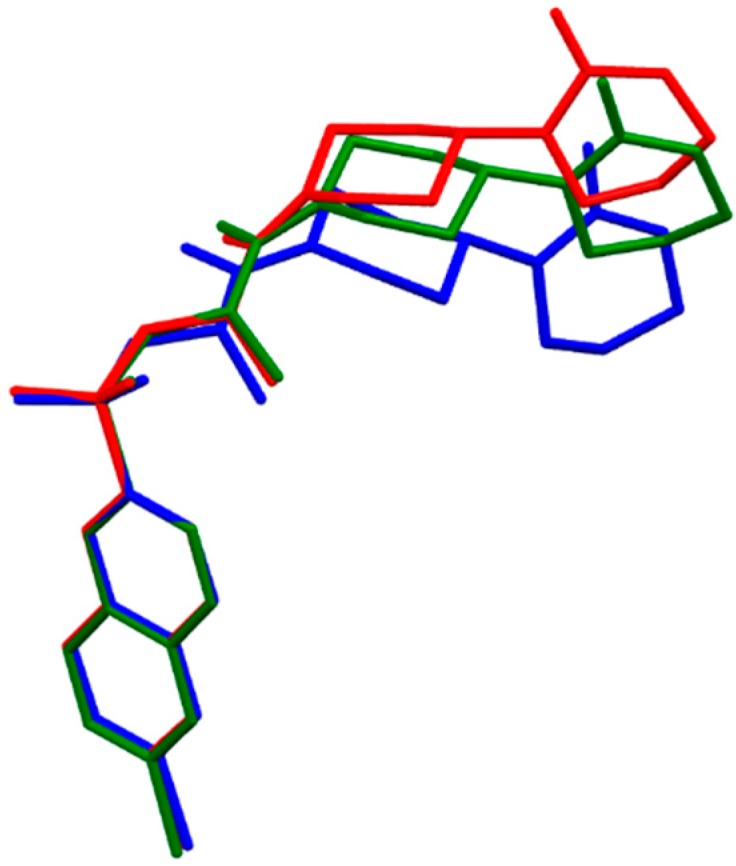
Molecular superimposition of the Becke3LYP optimized molecular structure of letaxaban (green), in solution optimized letaxaban (red) and letaxaban from the co-crystal with coagulation factor Xa, PDB.3KL6 (blue). For simplicity the hydrogen atoms are omitted.

The synclinal orientation of the hydroxyl group towards sulfonyl group (the dihedral angle γ[S(3)-C(4)-C(5)-O(6)] is about 73°) ensures additional hydrogen-bonded interactions of letaxaban with the nitrogen atom of the main chain Gly216 of the fXa receptor. The tetrahydropyrimidinone group is in an anticlinal position with respect to the piperidinyl ring (dihedral angle η[C(10)-C(11)-N(12)-C(13)]; Table 1) and is involved in hydrophobic interaction with the aromatic rings of Tyr99, Phe174, and Trp215 located in the S4 site of the receptor [24]. The 3D geometry of letaxaban in water, computed with the polarizable continuum method using the CPCM model, did not appreciably differ from the geometries computed for isolated molecules (Table 1). The stable conformation letaxaban when bound at the fXa receptor (PDB file 3KL6) is close to the 3D structure of isolated drug and/or solvated conformer and only small changes in geometry upon complexation were observed (Figure 5), and the biologically active conformer is 96 kJ/mol less stable than the unbound structure.

#### 2.1.6. Tanogitran (Dual)

Tanogitran (*N*-[(2*R*)-2-(2-{[(4-carbamimidoylphenyl)amino]methyl}-1-methyl-1*H*-benzimidazol-5-yl)-1-oxo-1-(1-pyrrolidinyl)-2-propanyl]glycine) is a dual inhibitor of thrombin and factor Xa. Tanogitran (FXa *K*_i_ = 26 nM, thrombin *K*_i_ = 2.7 nM [4,27] is a reversible dual inhibitor evaluated in phase II clinical trial studies in human model of endotoxin-induced coagulation [27]. Its discovery is closely related to the development of dabigatran, a single direct oral thrombin inhibitor [7]. Tanogitran, like dabigatran, contains a central substituted benzimidazole skeleton, an amino group of the linker between phenylamidine and benzimidazole rings and, at the opposite end of a molecule, a 5-propyl linker carrying aliphatic substituent glycine and pyrolidinne groups. In the absence of experimental structural data, the initial geometry of tanogitran was constructed on the basis of our previous studies of its predecessor, dabigatran [12]. In the thermodynamically stable structure of the isolated molecule, the phenylamidine and pyrrolidinyl end groups are perpendicularly arranged to the plane of the central benzimidazole scaffold and oriented *synperiplanar*. The polar glycine part is also almost perpendicular to the benzimidazole scaffold and oppositely oriented to the pyrrolidinyl substituent (Figure S1). The amidine group of the phenylamidine substituent is twisted out of the benzene ring by about −20° (dihedral angle α[N(1)-C(2)-C(3)-C(4)], Figure 1). The methylamino group of the linker between phenylamidine and benzimidazole is antiperiplanar to the aromatic ring (dihedral angle β[ C(5)-C(6)-N(7)-C(8)] = 165.5°, B3LYP calculation). However, the conformational structure of the molecular fragment connected by this spacer was described differently by the two DFT methods (dihedral angle γ[C(6)-N(7)-C(8)-C(9)] is about 79° (B3LYP) and 49° (B97D)). Thus, in the B97D optimized geometry, the phenylamidine and pyrrolidinyl substituents were more closely oriented to each other (Figure S1). The solvent effect did not appreciably change the general shape of the molecule (Table 1).

#### 2.1.7. SAR107375 (Dual)

SAR107375 (5-chlorothiophene-2-carboxylic acid [(S)-2-[2-methyl-3-(2-oxopyrrolidin-1-yl)benzenesulfonylamino]-3-(4-methylpiperazin-1-yl)-3-oxopropyl]amide) is a novel dual inhibitor discovered by Meneyrol *et al.* [28]. The 3D molecular structure optimized at the B3LYP level of theory represents a sandwich arrangement of the basic 4-methylpiperazine and benzenesulfonyl amino moieties connected via an oxopropyl amide linker (Figure S1). A third substituent, chlorothiophene carboxamide, is oriented away from the molecular scaffold and forms a strong interaction in the S1 pocket of the receptor. The sulfonamide linker is in a *gauche* arrangement between the aromatic ring and an S-N bond (dihedral angle ξ[N(11)-S(12)-C(13)-C(14)] is about 60°). The N-H and S=O groups are also in a mutual *gauche* conformation (dihedral angle μ[C(7)-N(11)-S(12)-C(13)], Table 1). The unique conformation of the oxopropylamide and sulfonamide moieties (dihedral angle σ[N(9)-C(8)-C(7)-N(11)]) is stabilized via hydrogen bond interactions of the N-H···O=C type in an *antiperiplanar* arrangement (Table 1, Figure S1). The oxopyrrolidine end group and aromatic ring are in a mutual *anticlinal* position (dihedral angle ρ[C(14)-C(15)-N(16)-C(17)]; Table 1). Almost the same equilibrium conformation of SAR107375 resulted from the geometry optimization by means of the B97D functional (Table 1). The equilibrium structure of the thiophene amide moiety is stabilized by means of non-bonded interaction between thiophene sulfur and neighboring oxygen of 2-amide group (Figure S1). In this molecule, the computed distance (about 2.95 Å) between non-bonded S···O atoms of the carbonyl amido group and the thiophene ring is clearly shorter than the sum of the corresponding van der Waals radii for oxygen and sulfur atoms (3.32 Å) [29]. This effect has been observed in a large number of organosulfur compounds controlling the conformation of small and large molecules [30,31].

### 2.2. Dissociation Constants

Several potent fXa inhibitors entered into clinical trials contain an acidic or a basic center, which may be ionized and/or protonated at physiological pH. Dissociation of a drug plays important part in both the partition and the binding of such drugs with their target enzyme. Table 3 contains the macroscopic p*K*a values of the studied anticoagulants. The p*K*a values in the condensed phase (water) were calculated using program SPARC [32,33]. Eribaxaban, and letaxaban are present in neutral undissociated form at pH 7.4. Edoxaban contains a basic N-5 nitrogen atom of 5-methylthiazolopyridine moiety and is partly protonated at pH 7.4. The structurally related 1,4-benzodiazepane moiety of darexaban possesses highly-basic N-4 nitrogen atom (p*K*a = 8.11), which is highly protonated at physiological pH. The dual inhibitor SAR107375 contains a basic methylpiperazine moiety with the N-4 nitrogen primary protonation site. The N-H group of the sulfonamide moiety is a weak acid (p*K*a = 8.35) slightly ionized at physiological p*H*. The direct fXa inhibitor fidexaban and dual fXa and thrombin inhibitor tanogitran possess both acidic (carboxyl) and basic (amidine) functionality. Both moieties are completely ionised at physiological pH and may exist, like the direct thrombin inhibitor dabigatran [12], as zwitterionic structures. Clinically approved direct fXa inhibitor edoxaban exists as partially dissociated molecules at blood pH. Lipid solubility is the rate limiting factor for drugs that are neutral or mainly undissociated at pH 7.4, thus the investigation into the lipophilicity and water-solubility of fXa and fIIa inhibitors is of primary importance to facilitate the partitioning of the drug in the lipid biomembrane and into the systemic circulation [34].

**Table 3:** The p*K*a values (at 25° C) of the anticoagulants investigated (SPARC/p*K*a).

Drug	p*K*a	p*K*a		% Ionized Form	
	Exp.	Acid Function ^a^	Basic Function ^b^	Acid Function	Basic Function
Edoxaban	6.7 (FDA label) ^c^	11.08	7.23	0	40
Eribaxaban		9.04		2	
Fidexaban		3.54	12.28	100	100
Darexaban		8.76	8.11	4	83
Letaxaban		12.93		0	
Tanogitran		1.98	11.53	100	100
SAR107375		8.35	6.52	10	11

^a^ most acidic; ^b^ most basic; ^c^ more details are in Drug Bank [35].

### 2.3. Lipophilicity and Solubility

The direct fXa and fIIa inhibitors, as well as their dual derivatives, were designed on the basis of knowing the basic molecular features of the target proteins. The two most important binding sites (S1 and S4) are hydrophobic in nature (S1 is a deep cleft and S4 is a strongly hydrophobic pocket), thus it is important to know the lipophilicity of designed inhibitors. The calculated log *P* and log *S* values using the ALOGPS 2.1 program are shown in Table 4. The lipophilicity predictions within this program are based on the use of E-state indicies and on the associative neural network approach [36]. As it is shown previously [11,12], this method performs quite well by predictions of experimentally known log *P* values of anticoagulants. A trend in calculated compound lipophilicity was observed: it was lowest for the dual inhibitors tanogitran and SAR107375 and increased for the clinically approved fXa inhibitor edoxaban. The experimental direct fXa inhibitors eribaxaban, fidexaban, and darexaban possess greater lipophilicity. However, their development was later disrupted. Fidexaban, darexaban, and tanogitran are almost completely ionized at pH 7.4 (Table 3). For drugs with ionizable functional groups, the effective lipophilicity is depends on pH, and the distribution coefficient log *D* will be different from log *P*. The log *D* values, calculated from the predicted log *P* (ALOGP method) and p*K*a (Sparc) using the equation log *D* = log *P* − log (1 + 10^pH−p*K*a^) for acids and log *D* = log *P* − log (1 + 10^p*K*a−pH^) for bases [37], are also presented in Table 4. The calculated log*D*_7.4_ values for the ionizable drugs were substantially lower. Aqueous solubility *S* is one of tne most important facors, which has impact on pharmacokinetic properties of oral drugs. The computed log *S* (the intrinsic solubility in the neutral state), is a measure of a drug’s solubility (*S*). The studied anticoagulants were slightly soluble in water; nevertheless their computed solubility, between 7 and 200 mg/L, is enough for fast absorption (Table 4 and Table 5). Fidexaban and tanogitran contain polar benzamidine and a carboxylate groups and in aqueous solution exhibit amphoteric properties. Because of their zwitterionic character, and log *D*_7.4_ = −0.79 and −3.84, they have no appreciable bioavailability after oral application.

**Table 4:** Calculated lipophilicity (log *P*) and solubility (log *S*) of the anticoagulants studied.

Drug	ALOGPS	Log *D*, (pH = 7.4)	ALOGPS, Solubility
Edoxaban	1.61		−4.68 (11.4 mg/L)
Eribaxaban	3.35		−4.83 (7.2 mg/L)
Fidexaban	3.07	−0.79	−4.34 (24.2 mg/L)
Darexaban	4.03	3.24	−4.21 (29.1 mg/L)
Letaxaban	1.55		−3.34 (0.2 g/L)
Tanogitran	1.58	−3.84	−3.54 (0.14 g/L)
SAR107375	1.15		−4.42 (21.4 mg/L)

**Table 5:** Calculated absorption (%ABS), polar surface area (PSA) and Lipinski parameters of the anticoagulants studied.

Drug	%ABS	Volume	PSA	NROTB	*n* ON Acceptors	*n* OHNH Donors	Formula Weight
Edoxaban	61.9	469.39	136.62	6	11 ^a^	3	548.07 ^a^
Eribaxaban	77.0	407.20	92.67	5	8	2	484.91
Fidexaban	54.6	440.66	157.60	9	11 ^a^	5	526.55 ^a^
Darexaban	76.5	438.49	94.13	6	8	3	474.61
Letaxaban	72.1	403.34	107.02	5	8	2	479.99
Tanogitran	57.5	437.41	149.36	9	10	6 ^a^	477.57
SAR107375	67.9	473.92	119.12	8	10	2	568.18 ^a^

^a^ Violation of the Rule of Five (Mw *>* 500).

### 2.4. Absorption, Polar Surface Area, and “Rule of Five” Properties

The studied anticoagulants were developed for oral administration, which is associated with high patient compliance. However, low intestinal absorption of designed anticoagulants may limit their clinical application [38]. Properties of drugs, such as membrane permeability or bioavailability, have often been related to molecular parameters such as molecular weight (*MW*), lipophilicity (log *P*), or counts of hydrogen bond acceptors and donors in a molecule. Orally absorbed novel anticoagulants tend to obey “Rule of Five” properties designed by Lipinski *et al.* [39]. This guideline is frequently used as a filter for drug-like properties. The calculated molecular descriptors (percentages of absorption (%ABS), molecular polar surface areas (PSA) and Lipinski parameters) are shown in Table 5. The magnitude of absorption is expressed by the percentage of absorption, which was evaluated according to the expression: %ABS = 109 − 0.345 PSA [40]. The PSA was calculated by the fragment-based method of Ertl and coworkers [41]. Edoxaban, fidexaban, and SAR107375 violated the “rule of five” (the molecular weight is too high), tanogitran, edoxaban and fidexaban also exhibited hydrogen bonding capacity over the limit, which is closely related to their highly polar character expressed by the large polar surface area of the studied drugs (Table 5). Fidexaban and tanogitran contain the highly basic arginine mimetic amidine group as the P1 moiety, which contributes to their poor oral bioavailability (Table 5). For the dual inhibitors tanogitran and SAR107375, the higher number of rotatable bonds in comparison with the clinically useful fXa inhibitors apixaban and rivaroxaban [11] impart higher conformational flexibility, enabling their interaction at the fXa and thrombin interaction sites in different conformations. Edoxaban, with a large polar surface area (136.62), may have worse absorption in comparison with rivaroxaban and apixaban [11]. Despite tremendous efforts connected with their discovery, the clinical development of eribaxaban, fidexaban, darexaban, and letaxaban has recently been halted [20,42]. The direct thrombin and fXa inhibitor tanogitran is a very flexible molecule with high total number of proton acceptor and proton donor groups (16), high PSA (149) and low absorption (Table 5). It contains, like the direct thrombin inhibitor dabigatran [12], ionizable acidic and basic functional groups, and under physiological conditions is present in the form of a charged species. Ionization of acidic or basic groups and the high PSA of tanogitran are not compatible with its oral application and tanogitran is not orally bioavailable. The dual inhibitor SAR107375 represents an improvement in structural, physicochemical and pharmacokinetic characteristics over tanogitran. At blood p*H*, SAR107375 predominantly exists in neutral form (Table 3). In contrast to tanogitran, it is better absorbed and more lipophilic and active after oral application (Table 5).

### 2.5. Selection Criteria for Drug-Like Properties of fXa Inhibitors

Extensive screening programs in medicinal chemistry offered large number of structurally different and novel direct fXa inhibitors with biochemical activity in nano molar range. However, the discovery of a clinically useful fXa inhibitor is a very difficult task because different physicochemical properties are needed for either the interaction of an inhibitor to the active site of fXa or its absorption from the gastrointestinal tract. Table 6 includes important molecular descriptors and measured binding affinities of 11 fXa inhibitors entered various stages of clinical trials. This Table includes two parenteral agents DX9065a and otamixaban, as well as, several orally active direct FXa inhibitors. 

**Table 6:** Calculated molecular descriptors and experimental biochemical activity (*K*i) of the novel anticoagulants.

Drug	%ABS	Volume Å^3^	PSA Å^2^	Clog *S*	Clog *P*	Formula Weight, Da	*K*i ^b^, nmol/L	Bioavailability ^c^ %
Rivaroxaban ^a^	77.8	351.74	88.18	−4.64	1.74	435.89	0.4	80
Apixaban ^a^	69.8	406.55	110.77	−3.83	2.23	459.51	0.08	50
Otamixaban ^a^	62.7	407.73	130.73	−5.35	2.12	446.51	0.4	
Betrixaban **^a^**	70.1	392.76	107.41	−4.44	2.86	451.91	0.117	47
Razaxaban ^a^	66.5	423.70	120.04	−4.07	3.90	528.47 (viol.)	0.19	
DX-9065a ^a^	65.3	410.68	123.50	−4.49	2.61	444.53	41	3
Edoxaban	61.9	469.39	136.62	−4.68	1.61	548.07 (viol.)	0.56	62
Eribaxaban	77.0	407.20	92.67	−4.83	3.35	484.91	0.32	
Fidexaban	54.6	440.66	157.60	−4.34	3.07	526.55 (viol)	0.11	
Darexaban	76.5	438.49	94.13	−4.21	4.03	474.61	31	
Letaxaban	72.1	403.34	107.02	−3.34	1.55	479.99	1.8	50

^a^ Reference [11]; ^b^ BindingDB database [43] and PDBbind-CN Database [44]; ^c^ Experimental bioavailability, references [45,46,47].

Three inhibitors approved for clinical use (apixaban, rivaroxaban and edoxaban) exhibit different physicochemical characteristics and also different absorption. The experimental bioavailability for oral drugs is different, but very high [45,46,47]. Within the limits of the examined property space, and with the exception of edoxaban, the percentage of experimental bioavailability seems to somehow correlate with calculated absorption (%ABS, Table 6). Computed molecular descriptors of these drugs were also used to determine selection criteria for drug-likeness of compounds (Table 7). Molecular weight, lipophilicity and solubility, together with the conformational structure discussed in the Section 2.1, represent important physicochemical properties of drug-like fXa inhibitors. The inflated size of molecules results in molecular weight frequently violating the “rule of five”. The lipophilicity within a range of log *P* between 1 and 4, and solubility log *S* between −3 and −5, to ensure optimal physicochemical properties [48,49], and correspond well to the absence of importance of the hydrophobic effect determined from QSAR studies [49]. Polar surface area, owing to a structurally heterogeneous character of inhibitors, is from relatively large interval of values (Table 7). Drugs like rivaroxaban, apixaban, darexaban, eribaxan, letaxaban and betrixaban with lowest PSA values exhibit largest absorption.

**Table 7:** Drug-like properties of the novel direct fxa inhibitors.

Molecular Weight	430–550
Octanol/water partition coefficient (clog *P*)	1–4
Aqueous solubility (clog *S*)	(−3.3)–(−5.3)
Polar surface area (PSA, Å^2^)	90–160
Volume (Vol, Å^3^)	350–470
Percent of oral absorption (%ABS)	55–76

## 3. Computational Details

Density functional theory (DFT) [50] calculations of edoxaban, eribaxaban, fidexaban, darexaban (YM150), letaxaban (TAK-442), tanogitran and SAR107375, (Figure 1) were carried out with the Gaussian 09 computer code [51] employing the Becke3LYP [52,53,54], Grimme’s B97D [16] functionals and using 6-31++G(d,p) basis set [55]. The geometry optimization calculations of these drugs were also investigated in aqueous solution. The hydration free energy was computed using the polarizable continuum method within conductor-like polarizable continuum model (CPCM) [56]. Lipophilicity and water solubility calculations were carried out using web-based VCCLAB [36]. Calculations of macroscopic p*K*a were carried out by means of the program SPARC [33] developed by Carreira *et al.* [32]. For calculations of molecular volume and polar surface area, the fragment-based method of Ertl and coworkers [41] was used.

## 4. Conclusions

In conclusion, the data presented in this theoretical study were able to determine the stable conformations, solvent effect, acidity, lipophilicity, solubility, absorption, and polar surface area of seven direct fXa inhibitors and two dual fXa and thrombin inhibitors for which a relatively small amount of experimental physicochemical data exist, considering their pharmacological importance. Using theoretical methods, the following conclusions can be drawn.

i) The fully optimized most stable conformers of these drugs possess a characteristic l-shaped structure. Examination of the spatial models of the B3LYP and B97D optimized structures indicated that the equilibrium geometries computed using the B3LYP and B97D functionals are in some cases different.
ii) Water had a remarkable effect on the geometry of the studied anticoagulants. The anticoagulant drugs exhibit considerable stability in this solvent, as expected.
iii) Eribaxaban, and letaxaban are present in neutral undissociated form at pH 7.4. Fidexaban and tanogitran exist as zwitterionic structures.
iv) A trend in the compound lipophilicity was also observed. It is lowest for the dual inhibitors tanogitran and SAR107375 and increase for the clinically approved fXa inhibitor edoxaban.
v) The studied anticoagulants were only slightly soluble in water, but their computed solubility between 7 and 200 mg/L is sufficient for fast absorption.
vi) The dual inhibitor SAR107375 represents an improvement in structural, physicochemical and pharmacokinetic characteristics over tanogitran. At blood p*H*, SAR107375 predominantly exists in neutral form. In contrast to tanogitran, it is better absorbed and more lipophilic and active after oral application.

## Supplementary Materials

The following are available online at: http://www.mdpi.com/1420-3049/21/2/185/s1. Figure S1: The geometries of the drugs studied. Molecule figures were generated using Mercury software., Table S1: Cartesian coordinates (Å) of the anticoagulants investigated, optimized at the B3LYP/6-31++G(d,p) level of the density functional theory.

## References

[1] Vermeer C., Schurgers L.J. (2000). A comprehensive review of vitamin K and vitamin K antagonists. Hematol. Oncol. Clin. N. Am..

[2] Becattini C., Vedovati M.C., Agnelli G. (2012). Old and new oral anticoagulants for venous thromboembolism and atrial fibrillation: A review of the literature. Thromb. Res..

[3] Scaglione F. (2013). New oral anticoagulants: Comparative pharmacology with vitamin K antagonists. Clin. Pharmacokinet..

[4] Pinto D.J.P., Smallheer J.M., Cheney D.L., Knabb R.M., Wexler R.R. (2010). Factor Xa inhibitors: Next-generation antithrombotic agents. J. Med. Chem..

[5] Levy J.H., Spyropoulos A.C., Samama C.M., Douketis J. (2014). Direct oral anticoagulants: New drugs and new concepts. JACC Cardiovasc. Interv..

[6] Schwienhorst A. (2006). Direct thrombin inhibitors—A survey of recent developments. Cell. Mol. Life Sci..

[7] Graefe-Mody E.U., Schühly U., Rathgen K., Stähle H., Leitner M.J., Jilma B. (2006). Pharmacokinetics and pharmacodynamics of BIBT 986, a novel small molecule dual inhibitor of thrombin and factor Xa. J. Thromb. Haemost..

[8] He L.W., Dai W.Ch., Li N.G. (2015). Development of orally active thrombin inhibitors for the treatment of thrombotic disorder diseases. Molecules.

[9] Giardino E.C., Haertlein B.J., de Garavilla L., Costanzo M.J., Damiano B.P., Andrade-Gordon P., Maryanoff B.E. (2010). Cooperative antithrombotic effect from the simultaneous inhibition of thrombin and factor Xa. Blood Coagul. Fibrinol..

[10] Nar H. (2012). The role of structural information in the discovery of direct thrombin and factor Xa inhibitors. Trends Pharmacol. Sci..

[11] Remko M. (2009). Molecular structure, lipophilicity, solubility, absorption, and polar surface area of novel anticoagulant agents. J. Mol. Struct..

[12] Remko M., Broer R., Remková A. (2014). A comparative study of the molecular structure, lipophilicity, solubility, acidity, absorption and polar surface area of coumarinic anticoagulants and direct thrombin inhibitors. RSC Adv..

[13] Sebastiani D., Röthlisberger U., Cavalli A., Folkers G., Recanatini M., Scapozza L., Rovira C., Raber J., Llano J., Eriksson L.A., Carloni P., Alber F. (2003). Quantum Medicinal Chemistry.

[14] Ooms F. (2000). Molecular modeling and computer aided drug design. Examples of their applications in medicinal chemistry. Curr. Med. Chem..

[15] Sliwoski G., Kothiwale S., Meiler J., Lowe E.W. (2014). Computational methods in drug discovery. Pharmacol. Rev..

[16] Grimme S. (2006). Semiempirical GGA-type density functional constructed with a long-range dispersion correction. J. Comput. Chem..

[17] Minor Ch., Tellor K.B., Armbruster A.L. (2015). Edoxaban, a novel oral factor Xa inhibitor. Ann. Pharmacother..

[18] Kahn R., Fourme R., André D., Renaud M. (1973). Crystal structure of cyclohexane I and II. Acta Crystallogr. B.

[19] Kohrt J.T., Bigge C.F., Bryant J.W., Casimiro-Garcia A., Chi L., Cody WL., Dahring T., Dudley D.A., Filipski K.J., Haarer S. (2007). The Discovery of (2*R*,4*R*)-*N*-(4-chlorophenyl)-*N*-(2-fluoro-4-(2-oxopyridin-1(2*H*)-yl)phenyl)-4-methoxypyrrolidine-1,2-dicarboxamide (PD 0348292), an orally efficacious factor Xa inhibitor. Chem. Biol. Drug Des..

[20] Ahrens I., Peter K., Lip G.Y., Bode C. (2012). Development and clinical applications of novel oral anticoagulants. Part II. Drugs under clinical investigation. Discov. Med..

[21] Phillips G.B., Buckman B.O., Davey D.D., Eagen K.A., Guilford W.J., Hinchman J., Ho E., Koovakkat S., Liang A., Light D.R. (1998). Discovery of *N*-[2-[5-[Amino(imino)methyl]-2-hydroxyphenoxy]-3,5-difluoro-6-[3-(4,5-dihydro-1-methyl-1*H*-imidazol-2-yl)phenoxy]pyridin-4-yl]-*N*-methylglycine (ZK-807834): A potent, selective, and orally active inhibitor of the blood coagulation enzyme factor Xa. J. Med. Chem..

[22] Adler M., Davey D.D., Phillips G.B., Kim S.H., Jancarik J., Rumennik G., Light D.R., Whitlow M. (2000). Preparation, characterization, and the crystal structure of the inhibitor ZK-807834 (CI-1031) complexed with factor Xa. Biochemistry.

[23] Hirayama F., Koshio H., Ishihara T., Hachiya S., Sugasawa K., Koga Y., Seki N., Shiraki R., Shigenaga T., Iwatsuki Y. (2011). Discovery of *N*-[2-Hydroxy-6-(4-methoxybenzamido)phenyl]-4-(4-methyl-1,4-diazepan-1-yl)benzamide (Darexaban, YM150) as a potent and orally available factor Xa inhibitor. J. Med. Chem..

[24] Fujimoto T., Imaeda Y., Konishi N., Hiroe K., Kawamura M., Textor G.P., Aertgeerts K., Kubo K. (2010). Discovery of a tetrahydropyrimidin-2(1*H*)-one derivative (TAK-442) as a potent, selective, and orally active factor Xa inhibitor. J. Med. Chem..

[25] Remko M., Herich P., Gregáň F., Kožíšek J. (2014). Structure, acidity and basicity of a benzene disulfonamide inhibitor of carbonic anhydrase. J. Mol. Struct..

[26] Remko M., Kožíšek J., Semanová J., Gregáň F. (2010). Synthesis, crystal and molecular structure of two biologically active aromatic sulfonamides and their hydrochloride salts. J. Mol. Struct..

[27] Leitner J.M., Jilma B., Mayr F.B., Cardona F., Spiel A.O., Firbas C., Rathgen K., Stähl H., Schühly U., Graefe-Mody E.U. (2007). Pharmacokinetics and pharmacodynamics of the dual FII/FX inhibitor BIBT 986 in endotoxin-induced coagulation. Clin. Pharmacol. Ther..

[28] Meneyrol J., Follmann M., Lassalle G., Wehner V., Barre G., Rousseaux T., Altenburger J.-M., Petit F., Bocskei Z., Schreuder H. (2013). 5-Chlorothiophene-2-carboxylic acid [(*S*)-2-[2-methyl-3-(2-oxopyrrolidin-1-yl)benzenesulfonylamino]-3-(4-methylpiperazin-1-yl)-3-oxopropyl]amide (SAR107375), a selective and potent orally active dual thrombin and factor Xa inhibitor. J. Med. Chem..

[29] Bondi A. (1964). Van der Waals volumes and radii. J. Phys. Chem..

[30] Remko M. (2010). Molecular structure, p*K*a, lipophilicity, solubility and absorption of biologically active aromatic and heterocyclic sulfonamides. J. Mol. Struct..

[31] Zhou F., Liu R., Li P., Zhang H. (2015). On the properties of S···O and S···π noncovalent interactions: The analysis of geometry, interaction energy and electron density. New J. Chem..

[32] Hilal S., Karickhoff S.W., Carreira L.A. (1995). A Rigorous test for SPARC’s chemical reactivity models: Estimation of more than 4300 ionization p*K*as. Quant. Struc. Act. Real..

[33] SPARC. http://archemcalc.com/sparc/.

[34] Malkia A., Murtomaki L., Urtti A., Kontturi K. (2004). Drug permeation in biomembranes: *In vitro* and *in silico* prediction and influence of physicochemical properties. Eur. J. Pharm. Sci..

[35] Drug Bank. http://www.drugbank.ca/drugs/DB09075.

[36] Tetko I.V., Gasteiger J., Todeschini R., Mauri A., Livingstone D., Ertl P., Palyulin V.A., Radchenko E.V., Zefirov N.S., Makarenko A.S. (2005). Virtual computational chemistry laboratory—Design and description. J. Comput. Aided Mol. Des..

[37] Xing L., Glen R.C. (2002). Novel Methods for the Prediction of logP, p*K*a, and logD. J. Chem. Inf. Comput. Sci..

[38] Veber D.F., Johnson R.S., Cheng H.Y., Smith B.R., Ward K.W., Kapple K.D. (2002). Molecular properties that influence the oral bioavailability of drug candidates. J. Med. Chem..

[39] Lipinski C.A., Lombardo F., Dominy B.W., Feeney P.J. (1997). Experimental and computational approaches to estimate solubility and permeability in drug discovery and development settings. Adv. Drug Deliv. Rev..

[40] Zhao Y.H., Abraham M.H., Lee J., Hersey A., Luscombe C.N., Beck G., Sherborne B. (2002). Cooper, Rate-limited steps of human oral absorption and QSAR studies. Pharm. Res..

[41] Ertl P., Rohde B., Selzer P. (2000). Fast calculation of molecular polar surface area as a sum of fragment-based contributions and its application to the prediction of drug transport properties. J. Med. Chem..

[42] Gómez-Outes A., Suárez-Gea M.L., Lecumberri R., Terleira-Fernández A.I., Vargas-Castrillón E., Rocha E. (2013). Potential role of new anticoagulants for prevention and treatment of venous thromboembolism in cancer patients. Vasc. Health Risk Manag..

[43] Liu T., Lin Y., Wen X., Jorrisen R.N., Gilson M.K. (2007). *BindingDB*: A web-accessible database of experimentally determined protein-ligand binding affinities. Nucleic Acids Res..

[44] Liu Z., Li Y., Han L., Li J., Liu J., Zhao Z., Nie W., Liu Y., Wang R. (2015). PDB-wide collection of binding data: Current status of the PDBbind database. Bioinformatics.

[45] Toschi V., Lettino M. (2011). Inhibitors of propagation of coagulation: Factors V and X. Br. J. Clin. Pharmacol..

[46] Masotti L., Campanini M. (2013). Pharmacology of new oral anticoagulants: Mechanism of action, pharmacokinetics, pharmacodynamics. Italian J. Med..

[47] Weitz J.I., Eikelboom J.W., Samama M.M. (2012). New antithrombotic drugs, antithrombotic therapy and prevention of thrombosis: American college of chest physicians, evidence-based clinical practice guidelines. Chest.

[48] Arnott J.A., Planey S.L. (2012). The influence of lipophilicity in drug discovery and design. Expert Opin. Drug Discov..

[49] Kontogiorgis C.A., Hadjipavlou-Litina D. (2004). Current trends in quantitative structure activity relationships on fxa inhibitors: Evaluation and comparative analysis. Med. Res. Rev..

[50] Bickelhaupt F.M., Baerends E.J., Lipkowitz K.B., Boyd D.B. (2000). Kohn-sham density functional theory: Predicting and understanding chemistry. Reviews in Computational Chemistry.

[51] (2011). Gaussian 09.

[52] Becke D. (1993). Density-functional thermochemistry. III. The role of exact exchange. J. Chem. Phys..

[53] Lee C., Yang W., Parr R.G. (1988). Development of the Colle-Salvetti correlation-energy formula into a functional of the electron density. Phys. Rev. B.

[54] Kohn W., Sham L.J. (1965). Self-consistent equations including exchange and correlation effects. Phys. Rev. A.

[55] Hehre W.J., Radom L., Schleyer P.V.R., Pople J.A. (1986). Ab Initio Molecular Orbital Theory.

[56] Cossi M., Rega N., Scalman I.G., Barone V. (2003). Energies, structures, and electronic properties of molecules in solution with the C-PCM solvation model. J. Comp. Chem..

