# Supplementary material for: Theoretical Study of Molecular Structure and Physicochemical Properties of Novel Factor Xa Inhibitors and Dual Factor Xa and Factor IIa Inhibitors
Source: Molecules. 2016 Feb 4;21(2):185. doi: 10.3390/molecules21020185 (PMC6273828; doi:10.3390/molecules21020185)
Supplement: Supplementary file 1 [file molecules-21-00185-s001.pdf]

Supplementary Materials: Theoretical Study of Molecular Structure and Physicochemical Properties of Novel Factor Xa Inhibitors and Dual Factor Xa and Factor IIa Inhibitors

Milan Remko [1,*], Anna Remková [2] and Ria Broer [3]

Table S1. Cartesian coordinates (Å) of the anticoagulants investigated, optimized at the B3LYP/6-31++G(d,p) level of the density functional theory.

Center Number	Atomic Number	Atomic Type	Coordinates (Angstroms)		
			X	Y	Z
		EDOXABAN			
1	17	0	−9.790233	1.395728	−0.474862
2	16	0	2.471313	3.170109	1.995877
3	8	0	4.036355	−5.126229	0.862375
4	8	0	1.478191	0.429080	2.871377
5	8	0	−2.673408	−3.155866	0.036393
6	8	0	−2.931681	0.358651	0.095830
7	7	0	1.602791	−0.562206	0.796993
8	7	0	−1.024058	−1.569566	0.254661
9	7	0	5.139078	−4.200280	−0.901355
10	7	0	3.624733	5.807317	−0.904822
11	7	0	2.655278	1.822693	−0.207408
12	7	0	−4.576451	−1.263879	−0.053141
13	7	0	−6.857607	−1.299629	−0.259923
14	6	0	1.218888	−1.911388	1.220316
15	6	0	0.113380	−2.479559	0.305310
16	6	0	2.973484	−3.215003	−0.126527
17	6	0	2.417734	−2.878687	1.270298
18	6	0	0.646659	−2.812927	−1.101636
19	6	0	1.846862	−3.768616	−1.032412
20	6	0	4.099297	−4.251850	−0.004208
21	6	0	1.755274	0.472367	1.674284
22	6	0	−2.294370	−1.985368	0.115529
23	6	0	2.290142	1.715466	1.044984
24	6	0	5.314287	−3.183554	−1.932540
25	6	0	6.247177	−5.140579	−0.754189
26	6	0	3.106665	3.090676	−0.481217
27	6	0	3.080328	3.962958	0.579344
28	6	0	3.588286	3.517525	−1.836937
29	6	0	3.534430	5.392477	0.497441
30	6	0	4.364234	4.837472	−1.718488
31	6	0	−3.300354	−0.813096	0.055293
32	6	0	4.165231	7.156390	−1.015032
33	6	0	−5.766956	−0.530521	−0.146896
34	6	0	−5.812927	0.873353	−0.124411
35	6	0	−7.060114	1.482359	−0.225954
36	6	0	−8.048926	−0.706582	−0.356335
37	6	0	−8.195591	0.681426	−0.343856
38	1	0	0.819133	−1.791304	2.230347
39	1	0	−0.248361	−3.408216	0.760057
40	1	0	3.363331	−2.292646	−0.572167
41	1	0	3.199730	−2.440795	1.899184

Table S1. *Cont.*

Center Number	Atomic Number	Atomic Type	Coordinates (Angstroms)		
			X	Y	Z
		**EDOXABAN**			
42	1	0	2.109346	−3.809460	1.756922
43	1	0	−0.161579	−3.263142	−1.687094
44	1	0	0.926963	−1.884309	−1.619180
45	1	0	1.525382	−4.737089	−0.628618
46	1	0	2.224153	−3.962039	−2.043669
47	1	0	1.955130	−0.394132	−0.137940
48	1	0	−0.872949	−0.566173	0.289080
49	1	0	5.912118	−2.332745	−1.576029
50	1	0	4.359051	−2.812393	−2.302379
51	1	0	5.839678	−3.633062	−2.780724
52	1	0	6.020582	−5.823296	0.062729
53	1	0	7.178531	−4.603572	−0.532296
54	1	0	6.386172	−5.711187	−1.680092
55	1	0	2.731202	3.648883	−2.509685
56	1	0	4.227385	2.740741	−2.272640
57	1	0	4.510544	5.499465	1.016779
58	1	0	2.828509	6.056300	1.010325
59	1	0	5.370325	4.642351	−1.294378
60	1	0	4.512156	5.270496	−2.712344
61	1	0	4.139241	7.478135	−2.060217
62	1	0	3.548992	7.848553	−0.432412
63	1	0	5.208510	7.236434	−0.652181
64	1	0	−4.675399	−2.277082	−0.078507
65	1	0	−4.902708	1.449744	−0.030466
66	1	0	−7.147591	2.563452	−0.213032
67	1	0	−8.915355	−1.355701	−0.446555
		**ERIBAXABAN**			
1	8	0	0.127578	4.226212	−0.809889
2	8	0	0.116250	1.817197	2.508821
3	8	0	−3.811063	1.480549	−1.654054
4	7	0	−2.140470	2.313171	−0.336920
5	6	0	−0.301256	1.805200	1.354437
6	6	0	−1.616088	2.542904	1.019283
7	1	0	−2.332763	2.218535	1.782072
8	6	0	−1.436613	4.079113	1.088487
9	1	0	−2.361877	4.537350	1.453317
10	1	0	−0.630994	4.368037	1.765838
11	6	0	−1.204557	4.487630	−0.376604
12	1	0	−1.454234	5.542737	−0.558130
13	6	0	−2.133056	3.551400	−1.146166
14	1	0	−3.146436	3.961838	−1.221881
15	1	0	−1.765690	3.358876	−2.155895
16	6	0	1.089801	5.183539	−0.387743
17	1	0	0.818791	6.192056	−0.732990
18	1	0	2.039254	4.892628	−0.840629
19	1	0	1.210016	5.199511	0.703878
20	6	0	−3.093433	1.352855	−0.665618
21	7	0	−3.134104	0.260795	0.185209
22	1	0	−2.409182	0.190902	0.884194
23	6	0	−3.989178	−0.860839	0.121564
24	6	0	−3.783195	−1.873017	1.074419

Table S1. *Cont.*

Center Number	Atomic Number	Atomic Type	Coordinates (Angstroms)		
			X	Y	Z
**ERIBAXABAN**					
25	6	0	−5.021361	−1.007245	−0.818437
26	6	0	−4.585789	−3.010618	1.096932
27	1	0	−2.988378	−1.772504	1.810027
28	6	0	−5.826106	−2.148106	−0.793268
29	1	0	−5.184621	−0.238741	−1.560402
30	6	0	−5.608926	−3.142418	0.158207
31	1	0	−4.416622	−3.784715	1.837200
32	1	0	−6.622501	−2.258260	−1.521277
33	17	0	−6.629809	−4.575961	0.177918
34	7	0	0.293385	1.161318	0.307207
35	1	0	−0.145684	1.312985	−0.595117
36	6	0	1.517274	0.474729	0.293431
37	6	0	1.960123	−0.031658	−0.935177
38	6	0	2.335009	0.253719	1.414170
39	6	0	3.150034	−0.719895	−1.086764
40	6	0	3.534336	−0.445720	1.286494
41	1	0	2.020922	0.637809	2.374456
42	6	0	3.942857	−0.936772	0.044974
43	1	0	3.450335	−1.059134	−2.071846
44	1	0	4.162219	−0.595169	2.155917
45	6	0	6.397658	−1.055525	0.305928
46	6	0	5.121754	−2.940367	−0.634824
47	6	0	7.574966	−1.869966	0.074441
48	6	0	6.248509	−3.680428	−0.842195
49	1	0	4.129470	−3.306757	−0.870466
50	6	0	7.505554	−3.118267	−0.475221
51	1	0	8.517039	−1.416333	0.360252
52	1	0	6.170260	−4.674676	−1.264172
53	1	0	8.415253	−3.691704	−0.632206
54	7	0	5.167948	−1.677832	−0.089626
55	8	0	6.400492	0.075589	0.788288
56	9	0	1.176545	0.181784	−2.030853
**FIDEXABAN**					
1	6	0	2.655411	1.793148	−0.612812
2	9	0	3.882815	2.305902	−0.362301
3	6	0	1.522620	2.627714	−0.635108
4	6	0	0.308196	1.940235	−0.859619
5	9	0	−0.845287	2.643449	−0.967137
6	6	0	0.272463	0.559349	−1.011753
7	7	0	1.372351	−0.186574	−1.002324
8	6	0	2.533423	0.416989	−0.817430
9	8	0	4.856998	−1.890721	−2.692893
10	8	0	3.698762	−0.302869	−0.820805
11	6	0	3.667643	−1.685410	−0.607592
12	6	0	3.127817	−2.260186	0.530358
13	6	0	3.196281	−3.648952	0.710228
14	6	0	3.842891	−4.426775	−0.264866
15	6	0	4.401213	−3.840181	−1.399278
16	6	0	4.313130	−2.458961	−1.583050
17	6	0	2.591533	−4.240330	1.938698

Table S1. *Cont.*

Center Number	Atomic Number	Atomic Type	Coordinates (Angstroms)		
			X	Y	Z
**FIDEXABAN**					
18	7	0	2.411565	−3.496473	2.973691
19	7	0	2.310677	−5.602712	1.865626
20	8	0	−0.905736	−0.073046	−1.303389
21	6	0	−1.898207	−0.154360	−0.334432
22	6	0	−1.615079	−0.163534	1.032889
23	6	0	−2.673387	−0.286429	1.937088
24	6	0	−3.986315	−0.423668	1.486770
25	6	0	−4.255769	−0.431241	0.107955
26	6	0	−3.203605	−0.286325	−0.803271
27	6	0	−5.644581	−0.521580	−0.409840
28	7	0	−6.598535	−1.363216	0.186203
29	6	0	−6.255548	−2.681099	0.708374
30	7	0	−6.059087	0.227869	−1.372819
31	6	0	−7.771353	−1.316579	−0.705582
32	6	0	−7.496402	−0.041918	−1.532903
33	7	0	1.562876	4.009608	−0.501676
34	6	0	0.662404	4.680854	0.421053
35	6	0	1.125237	4.768195	1.877092
36	8	0	0.720098	5.600071	2.658813
37	8	0	2.003177	3.796844	2.214252
38	6	0	2.767062	4.764495	−0.853710
39	1	0	4.744623	−0.929493	−2.629300
40	1	0	2.647242	−1.653146	1.287857
41	1	0	3.951198	−5.497353	−0.125129
42	1	0	4.915433	−4.435295	−2.146380
43	1	0	1.956327	−4.009498	3.731876
44	1	0	2.054451	−5.975755	0.961634
45	1	0	1.771612	−5.987023	2.629591
46	1	0	−0.592804	−0.087536	1.387150
47	1	0	−2.465049	−0.279391	3.002860
48	1	0	−4.801996	−0.497434	2.198284
49	1	0	−3.400760	−0.262477	−1.868624
50	1	0	−5.335804	−2.637595	1.291075
51	1	0	−6.122383	−3.418786	−0.100870
52	1	0	−7.059992	−3.032238	1.361915
53	1	0	−8.704264	−1.282552	−0.134499
54	1	0	−7.788501	−2.217984	−1.341185
55	1	0	−7.758214	−0.149598	−2.589838
56	1	0	−8.056618	0.818721	−1.142963
57	1	0	−0.314728	4.192819	0.431966
58	1	0	0.493573	5.707767	0.084627
59	1	0	2.218178	3.913832	3.155810
60	1	0	2.457748	5.777850	−1.128699
61	1	0	3.249453	4.311815	−1.720260
62	1	0	3.495717	4.823738	−0.036219
**DAREXABAN**					
1	8	0	−0.810778	−3.773743	−1.125646
2	8	0	−2.907706	−4.807588	−0.038439
3	8	0	−2.196508	0.161957	1.617031
4	8	0	−1.980295	6.308802	0.069644
5	7	0	4.649820	−1.101333	0.917010

Table S1. *Cont.*

Center Number	Atomic Number	Atomic Type	Coordinates (Angstroms)		
			X	Y	Z
		**DAREXABAN**			
6	7	0	5.206731	1.227930	−0.698893
7	7	0	−1.750861	−2.147966	0.173548
8	7	0	−3.549561	0.064801	−0.226818
9	6	0	5.822602	−1.703430	0.284020
10	6	0	6.269628	−1.013277	−1.014146
11	6	0	4.927667	0.168049	1.576377
12	6	0	6.470441	0.508458	−0.899051
13	6	0	4.799338	1.426381	0.689179
14	6	0	3.357524	−1.475477	0.585765
15	6	0	5.122652	2.477087	−1.444727
16	6	0	2.235535	−0.810671	1.142043
17	6	0	3.086170	−2.556069	−0.291040
18	6	0	0.937426	−1.215094	0.861815
19	6	0	1.785133	−2.952679	−0.561276
20	6	0	0.677635	−2.299288	0.004694
21	6	0	−0.674388	−2.796303	−0.358146
22	6	0	−3.125280	−2.392430	−0.070811
23	6	0	−4.004649	−1.291212	−0.179046
24	6	0	−3.671651	−3.699871	−0.147312
25	6	0	−5.381884	−1.487082	−0.333359
26	6	0	−5.052358	−3.874672	−0.310940
27	6	0	−5.903079	−2.778310	−0.395243
28	6	0	−2.747747	0.728729	0.662343
29	6	0	−2.566088	2.192497	0.421893
30	6	0	−2.198831	2.994902	1.518387
31	6	0	−2.716282	2.801863	−0.830207
32	6	0	−2.017823	4.361107	1.372137
33	6	0	−2.527014	4.176550	−0.994905
34	6	0	−2.183209	4.963981	0.112767
35	6	0	−2.133523	6.988898	−1.172981
36	1	0	5.638829	−2.765426	0.107331
37	1	0	6.638324	−1.657567	1.017051
38	1	0	5.530636	−1.193545	−1.802987
39	1	0	7.214437	−1.474272	−1.335171
40	1	0	5.948860	0.097811	1.961376
41	1	0	4.287866	0.292031	2.456365
42	1	0	6.906407	0.858221	−1.840173
43	1	0	7.213341	0.747880	−0.113672
44	1	0	3.758250	1.768636	0.671316
45	1	0	5.389675	2.227115	1.183614
46	1	0	5.843025	3.244833	−1.098309
47	1	0	4.115312	2.894950	−1.344736
48	1	0	5.302802	2.290998	−2.507691
49	1	0	2.370757	0.031264	1.808934
50	1	0	3.893711	−3.089956	−0.775518
51	1	0	0.132813	−0.658479	1.332274
52	1	0	1.602002	−3.783977	−1.233429
53	1	0	−1.595628	−1.377831	0.823727
54	1	0	−6.036598	−0.622730	−0.397736
55	1	0	−4.094535	0.651734	−0.842670
56	1	0	−5.425977	−4.891558	−0.364151
57	1	0	−6.972367	−2.925694	−0.511161
58	1	0	−2.063050	2.521801	2.484979

Table S1. *Cont.*

Center Number	Atomic Number	Atomic Type	Coordinates (Angstroms)		
			X	Y	Z
		**DAREXABAN**			
59	1	0	−2.943390	2.208755	−1.711812
60	1	0	−2.028076	−4.615310	−0.467254
61	1	0	−1.746763	4.986721	2.216140
62	1	0	−2.636037	4.611642	−1.980769
63	1	0	−3.153842	6.883301	−1.561022
64	1	0	−1.933065	8.039394	−0.961073
65	1	0	−1.416009	6.623985	−1.917811
		**LETAXABAN**			
1	6	0	−1.025454	−2.586986	0.618863
2	6	0	−3.339840	−1.258352	−0.595161
3	6	0	−3.808286	0.868928	−1.622802
4	6	0	−6.588471	2.491414	0.265877
5	6	0	−6.866769	1.518701	1.257249
6	6	0	−5.098679	0.118602	0.334848
7	6	0	3.803346	−1.913836	1.420884
8	6	0	4.969531	−0.146856	0.037465
9	6	0	3.008815	−1.465120	−0.857558
10	6	0	5.067102	2.363862	0.389858
11	8	0	−0.106500	−0.532841	−0.160679
12	6	0	0.202342	−1.921599	0.022464
13	16	0	−2.403231	−2.800541	−0.569624
14	8	0	−1.815664	−2.992539	−1.912774
15	8	0	−3.277707	−3.827104	0.029373
16	6	0	−3.070422	−0.292047	−1.593880
17	6	0	−4.832244	1.111275	−0.664172
18	6	0	−5.602758	2.305017	−0.675838
19	17	0	−7.539311	3.968699	0.252968
20	6	0	−6.131658	0.355270	1.282929
21	6	0	−4.330885	−1.075258	0.343067
22	6	0	1.395013	−2.085728	0.997649
23	8	0	1.191324	−2.407494	2.168488
24	7	0	2.650823	−1.835622	0.513257
25	6	0	4.563003	−0.580492	1.456342
26	6	0	3.740447	−0.112668	−0.890891
27	7	0	5.729012	1.108838	0.023403
28	6	0	6.087243	3.395307	0.868585
29	6	0	7.144550	3.598777	−0.212131
30	7	0	7.656081	2.297443	−0.619269
31	6	0	6.978334	1.099086	−0.580294
32	8	0	7.493814	0.072918	−1.036054
33	1	0	−1.404413	−2.041690	1.484948
34	1	0	−0.804809	−3.611622	0.926873
35	1	0	−3.618383	1.615072	−2.389312
36	1	0	−7.653710	1.700128	1.980593
37	1	0	4.468077	−2.714933	1.066684
38	1	0	3.430617	−2.190084	2.406388
39	1	0	5.666062	−0.891216	−0.358518
40	1	0	2.125425	−1.447047	−1.496616
41	1	0	3.669466	−2.243956	−1.265718
42	1	0	4.345073	2.156469	1.184409
43	1	0	4.501133	2.773754	−0.462032

Table S1. *Cont.*

Center Number	Atomic Number	Atomic Type	Coordinates (Angstroms)		
			X	Y	Z
		**LETAXABAN**			
44	1	0	0.511810	−0.138478	−0.788405
45	1	0	0.410871	−2.398126	−0.941978
46	1	0	−2.300555	−0.490014	−2.329986
47	1	0	−5.410046	3.062975	−1.427861
48	1	0	−6.338096	−0.398961	2.036970
49	1	0	−4.541592	−1.850111	1.073804
50	1	0	5.459936	−0.690417	2.075862
51	1	0	3.922047	0.172154	1.932492
52	1	0	4.058033	0.102186	−1.917818
53	1	0	3.046933	0.680268	−0.579739
54	1	0	6.561455	3.041431	1.790873
55	1	0	5.583573	4.342575	1.086721
56	1	0	7.976199	4.202370	0.166724
57	1	0	6.707658	4.139477	−1.066295
58	1	0	8.528103	2.236645	−1.124228
		**TANOGITRAN**			
1	6	0	2.308429	−0.540568	0.750781
2	6	0	2.039984	−1.335393	1.891249
3	6	0	0.914521	−2.151163	1.986258
4	6	0	0.046281	−2.166550	0.891540
5	6	0	0.289792	−1.385982	−0.260347
6	6	0	1.428718	−0.574210	−0.334937
7	7	0	−1.131800	−2.849577	0.631927
8	6	0	−1.544177	−2.442224	−0.624773
9	7	0	−0.725821	−1.577346	−1.184154
10	6	0	−2.786267	−2.956336	−1.304388
11	7	0	−4.013045	−2.773034	−0.534549
12	6	0	−4.687592	−1.550666	−0.477489
13	6	0	−6.008143	−1.522093	0.020686
14	6	0	−6.706664	−0.329249	0.120446
15	6	0	−6.118307	0.889553	−0.263078
16	6	0	−4.809591	0.856196	−0.763051
17	6	0	−4.095303	−0.338924	−0.873074
18	6	0	−6.904111	2.148179	−0.140213
19	7	0	−8.188910	2.088621	−0.075486
20	7	0	−6.155677	3.325530	−0.155519
21	6	0	−1.785374	−3.777765	1.540574
22	1	0	2.735589	−1.325122	2.722133
23	1	0	0.737477	−2.746682	2.876641
24	1	0	1.613051	0.005873	−1.234127
25	1	0	−2.826772	−2.462464	−2.283717
26	1	0	−2.681981	−4.031862	−1.490343
27	1	0	−4.629156	−3.573555	−0.541334
28	1	0	−6.481611	−2.451018	0.331613
29	1	0	−7.724814	−0.315678	0.493440
30	1	0	−4.336056	1.768016	−1.114527
31	1	0	−3.085843	−0.319398	−1.267703
32	1	0	−8.603839	3.015455	0.044147
33	1	0	−6.657229	4.172027	0.076583
34	1	0	−5.224619	3.287091	0.236093
35	1	0	−2.426689	−3.251603	2.255145
36	1	0	−2.398851	−4.480602	0.977131
37	1	0	−1.023553	−4.342491	2.084308

Table S1. *Cont.*

Center Number	Atomic Number	Atomic Type	Coordinates (Angstroms)		
			X	Y	Z
		**TANOGITRAN**			
38	6	0	3.610571	0.281902	0.655557
39	6	0	4.145764	0.735754	2.033594
40	1	0	4.574038	−0.121052	2.560192
41	1	0	4.932975	1.486688	1.918047
42	1	0	3.364842	1.165390	2.660946
43	7	0	4.604509	−0.620927	0.057265
44	1	0	4.273140	−0.954862	−0.844364
45	6	0	5.963184	−0.121547	−0.102617
46	1	0	6.020811	0.892844	−0.530518
47	1	0	6.495434	−0.107342	0.854331
48	6	0	6.734530	−1.011726	−1.049701
49	8	0	6.258289	−1.823157	−1.813354
50	8	0	8.069316	−0.771022	−0.977805
51	1	0	8.495038	−1.334708	−1.645662
52	6	0	3.366202	1.533011	−0.269558
53	8	0	3.893932	1.575621	−1.386131
54	6	0	2.355098	3.692929	−0.781052
55	6	0	1.706093	2.656042	1.332086
56	6	0	1.386974	4.612287	−0.023833
57	1	0	1.920644	3.309482	−1.712069
58	1	0	3.302816	4.172074	−1.041770
59	6	0	0.617741	3.643322	0.888973
60	1	0	2.255287	3.058646	2.194269
61	1	0	1.308382	1.675753	1.600006
62	1	0	1.943414	5.337037	0.582541
63	1	0	0.738360	5.173957	−0.701724
64	1	0	0.134695	4.134249	1.738995
65	1	0	−0.153977	3.113679	0.318155
66	7	0	2.585970	2.568154	0.151897
		**SAR107375**			
1	6	0	−5.007499	−2.009421	0.336803
2	6	0	−6.144304	−2.133698	1.181651
3	6	0	−6.725655	−0.913975	1.422821
4	17	0	−8.127171	−0.624613	2.396238
5	16	0	−5.911574	0.399500	0.631435
6	6	0	−4.745661	−0.711715	−0.044487
7	6	0	−3.688081	−0.132870	−0.904516
8	8	0	−3.688582	1.070503	−1.186599
9	7	0	−2.742039	−0.998511	−1.376547
10	6	0	−1.632848	−0.556422	−2.200005
11	6	0	−0.309437	−0.415790	−1.403290
12	6	0	−0.398447	0.605212	−0.242416
13	8	0	−0.411969	0.177508	0.919991
14	7	0	−0.466780	1.928276	−0.534058
15	6	0	−0.502289	2.522794	−1.873034
16	6	0	0.390367	3.765095	−1.924359
17	7	0	0.013332	4.724522	−0.891824
18	6	0	0.773238	5.963650	−0.991617
19	6	0	0.117978	4.116574	0.432978
20	6	0	−0.777439	2.884500	0.537751
21	7	0	0.021016	−1.718031	−0.810812
22	16	0	1.280975	−2.623582	−1.444253

Table S1. *Cont.*

Center Number	Atomic Number	Atomic Type	Coordinates (Angstroms)		
			X	Y	Z
		**SAR107375**			
23	8	0	1.304240	−3.864737	−0.665289
24	8	0	1.067226	−2.643006	−2.897601
25	6	0	2.814511	−1.692203	−1.154624
26	6	0	3.269633	−1.393059	0.148750
27	6	0	2.577181	−1.876215	1.401924
28	6	0	3.514645	−1.305628	−2.300496
29	6	0	4.706046	−0.592692	−2.170927
30	6	0	5.160565	−0.241777	−0.903952
31	6	0	4.446059	−0.619234	0.240169
32	7	0	4.946122	−0.204551	1.510305
33	6	0	4.249601	0.764374	2.371418
34	6	0	5.356900	1.249762	3.332620
35	6	0	6.363351	0.089426	3.357705
36	6	0	6.175457	−0.601399	2.008795
37	8	0	6.946975	−1.376209	1.465790
38	1	0	−4.418235	−2.864590	0.024058
39	1	0	−6.514622	−3.066911	1.587128
40	1	0	−2.694145	−1.937614	−1.008779
41	1	0	−1.926597	0.387000	−2.659613
42	1	0	−1.454303	−1.294334	−2.987036
43	1	0	0.473472	−0.116391	−2.108939
44	1	0	−1.538051	2.793195	−2.114632
45	1	0	−0.147319	1.807591	−2.614835
46	1	0	0.274796	4.237903	−2.905838
47	1	0	1.451543	3.455852	−1.823919
48	1	0	0.619534	6.412775	−1.977718
49	1	0	1.860919	5.815019	−0.844878
50	1	0	0.420123	6.672913	−0.236699
51	1	0	−0.199693	4.849167	1.182840
52	1	0	1.165307	3.832362	0.664770
53	1	0	−0.639298	2.378560	1.492552
54	1	0	−1.830498	3.179190	0.440616
55	1	0	0.027438	−1.669138	0.210604
56	1	0	2.124052	−2.855940	1.246493
57	1	0	1.792258	−1.178956	1.721963
58	1	0	3.300211	−1.964389	2.215291
59	1	0	3.126168	−1.578860	−3.274620
60	1	0	5.267903	−0.305875	−3.054064
61	1	0	6.078740	0.322502	−0.782354
62	1	0	3.423792	0.288190	2.914139
63	1	0	3.829265	1.572967	1.765260
64	1	0	4.957293	1.503168	4.317624
65	1	0	5.832276	2.147516	2.923572
66	1	0	7.404352	0.394306	3.482319
67	1	0	6.136908	−0.639581	4.145977

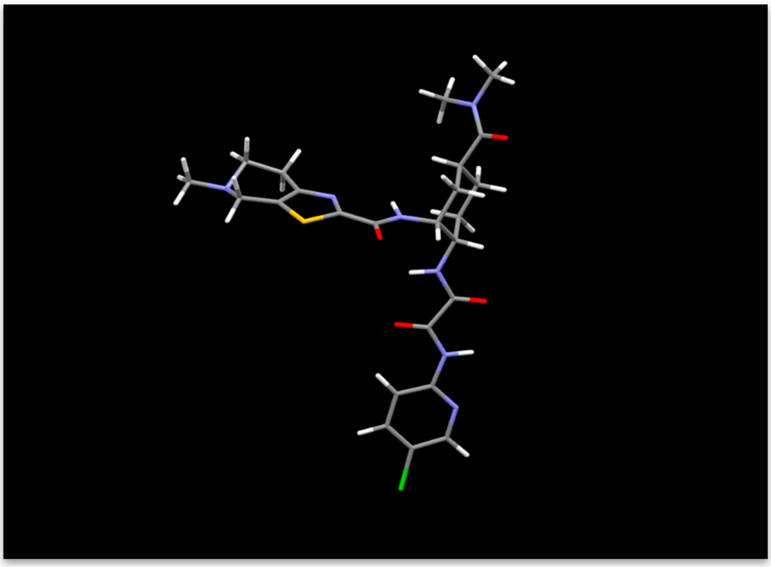

(**a**)

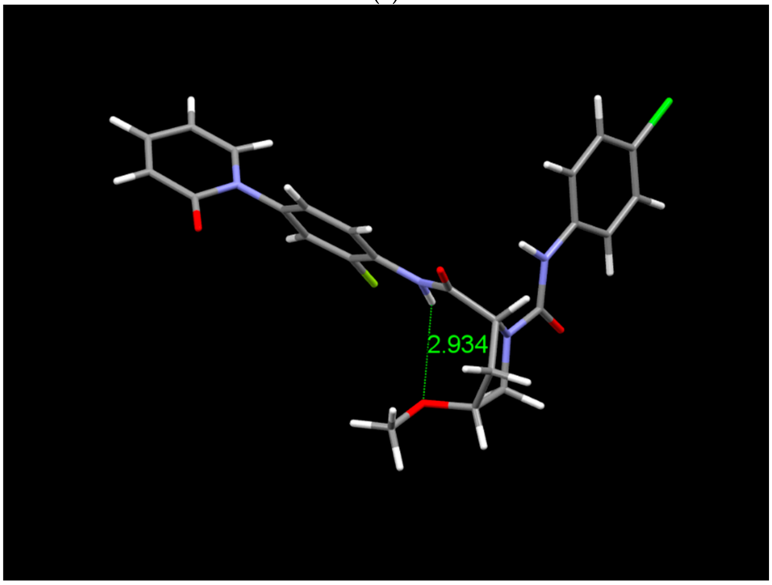

(**b**)

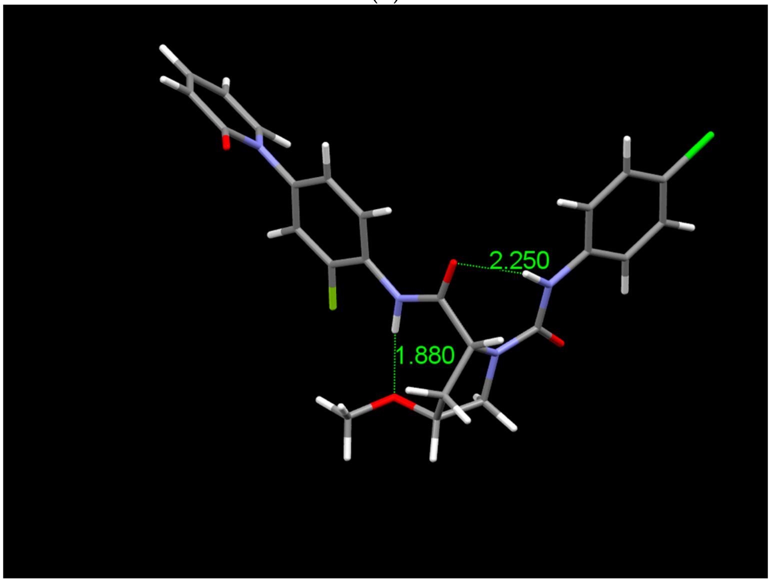

(**c**)

Figure S1. *Cont.*

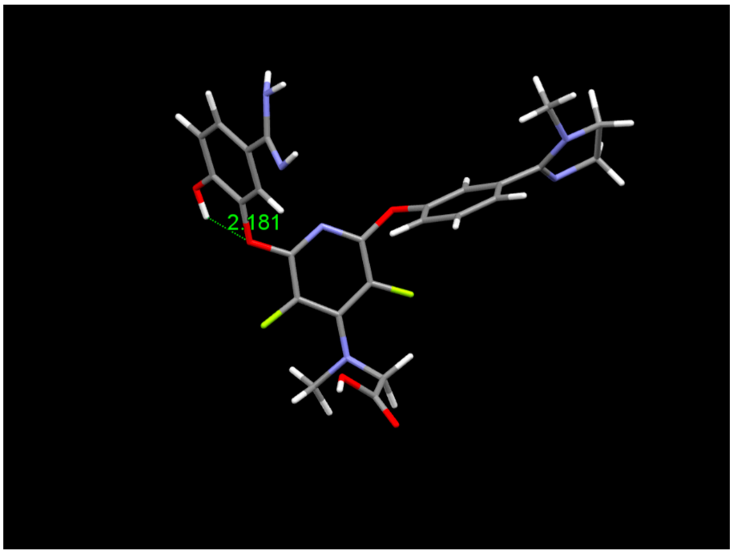

(**d**)

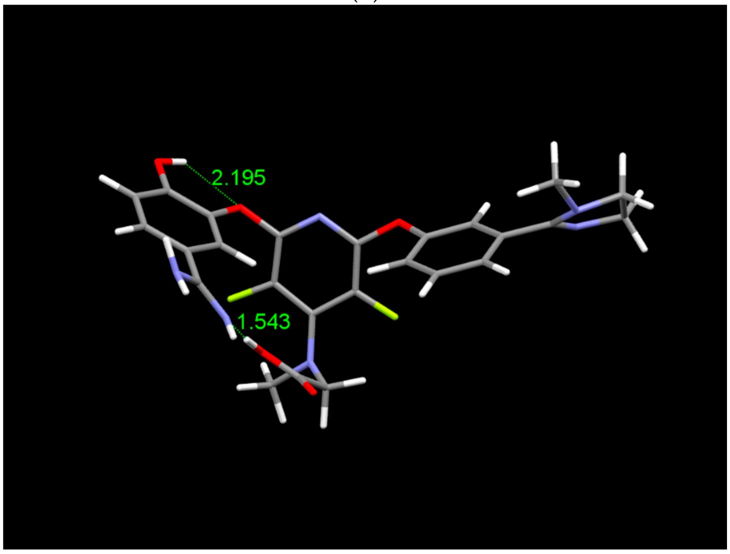

(**e**)

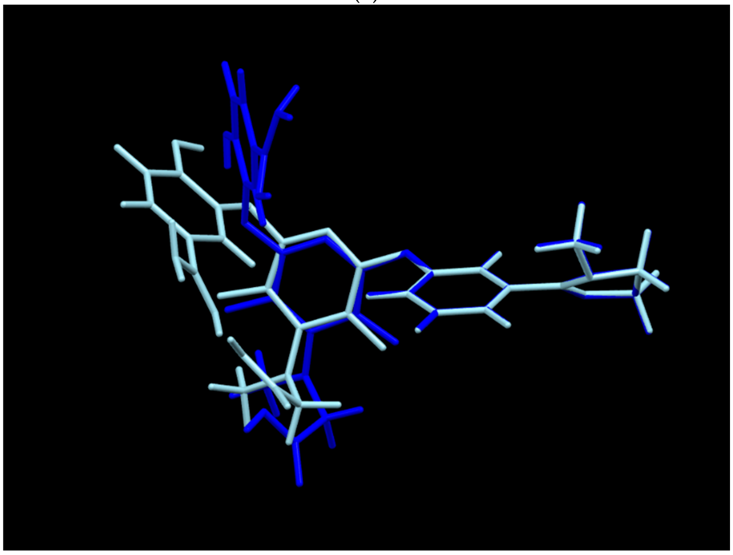

(**f**)

Figure S1. *Cont.*

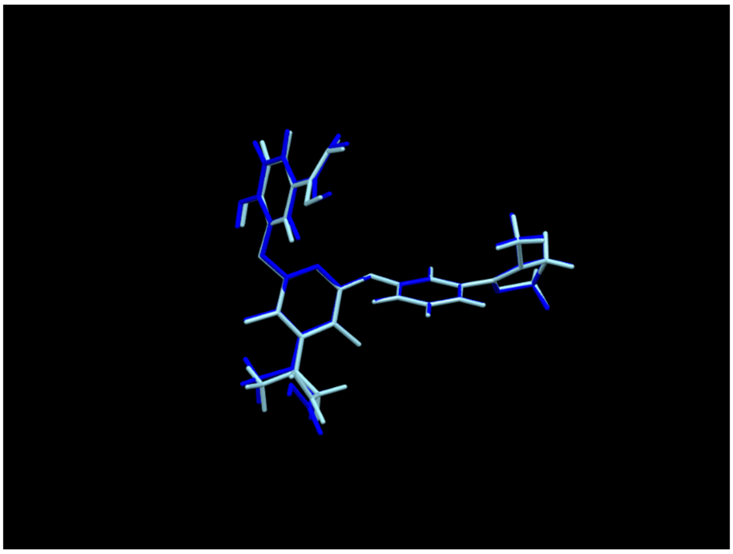

(**g**)

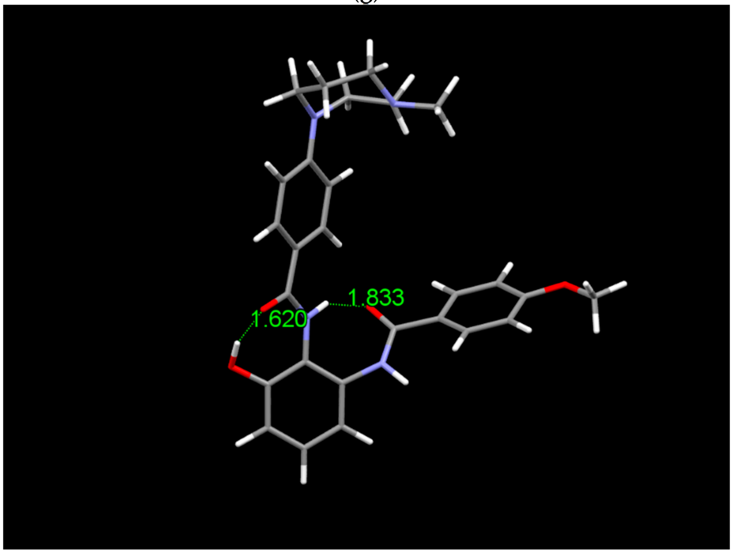

(**h**)

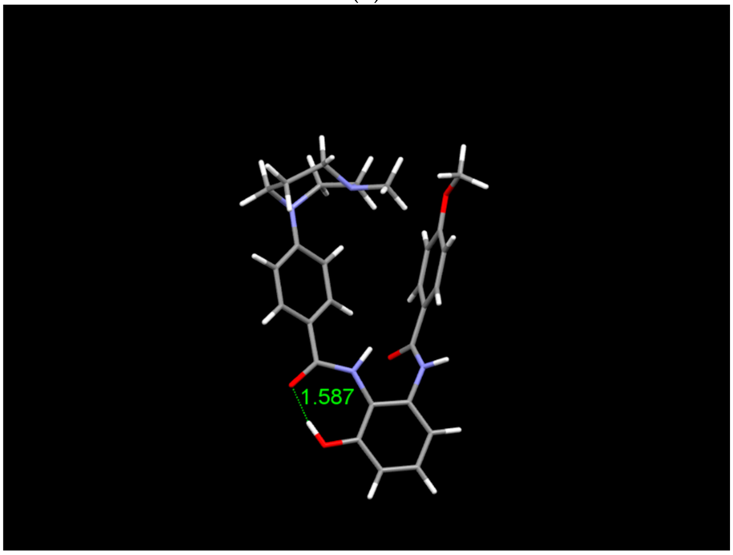

(**i**)

Figure S1. *Cont.*

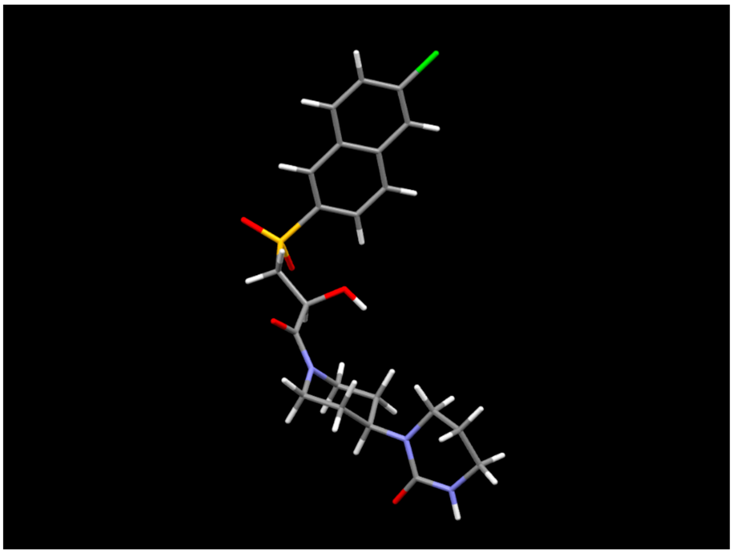

(**j**)

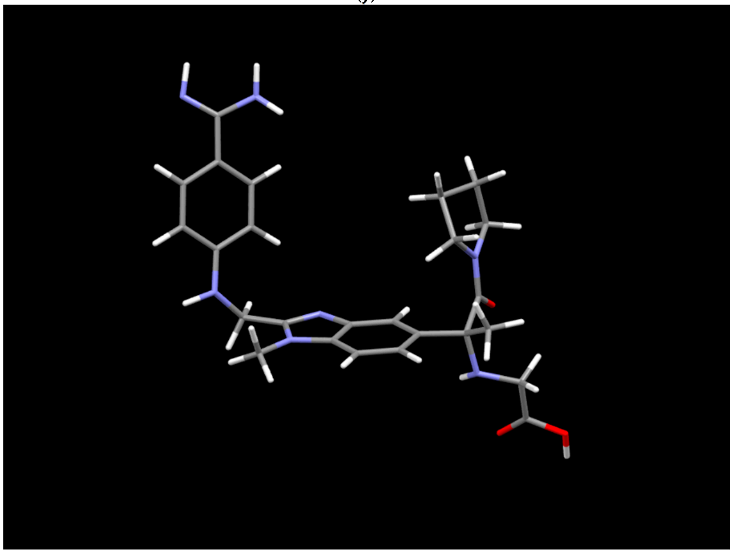

(**k**)

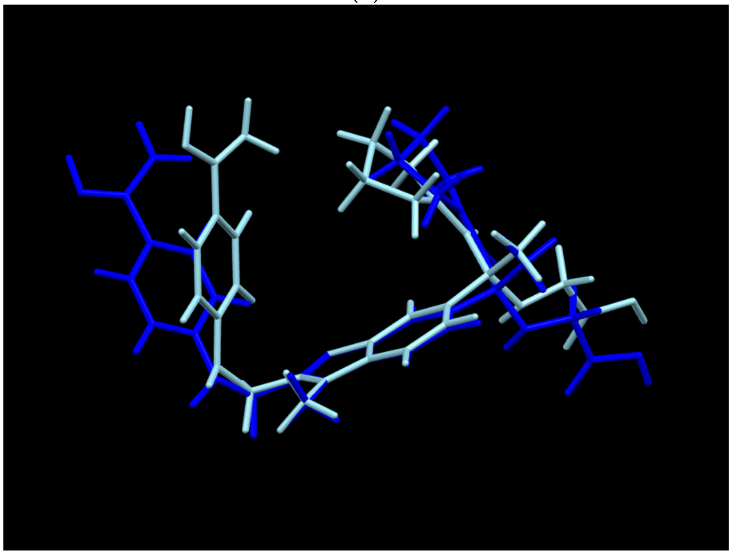

(**l**)

Figure S1. *Cont.*

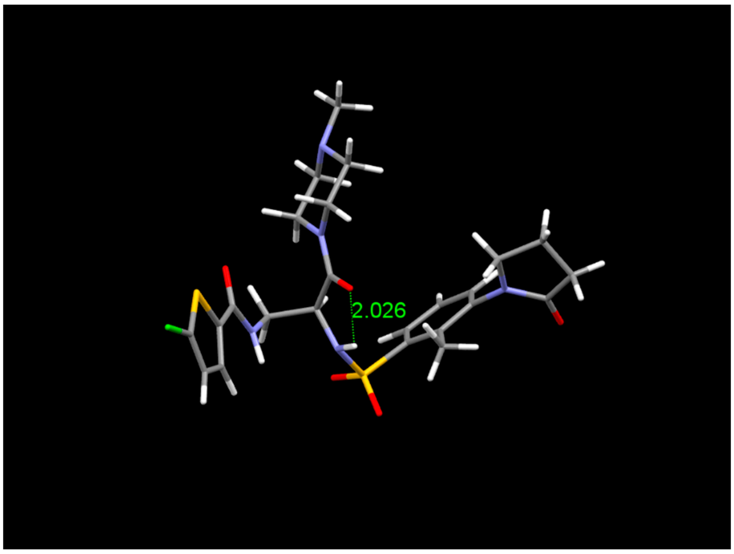

(**m**)

Figure S1. The geometries of the drugs studied. Molecule figures were generated using the Mercury software. Bond lengths are in Angstroms. (**a**) Overall shape of **edoxaban** computed at the Becke3LYP/6-31++G(d,p) level of theory; (**b**) Overall shape of **eribaxaban** computed at the Becke3LYP/6-31++G(d,p) level of theory; (**c**) Overall shape of **eribaxaban** computed at the B97D/6-31++G(d,p) level of theory; (**d**) Overall shape of **fidexaban** computed at the Becke3LYP/6-31++G(d,p) level of theory; (**e**) Overall shape of the **fidexaban** computed at the B97D/6-31++G(d,p) level of theory; (**f**) Molecular superimposition of the Becke3LYP optimized molecular structure of **fidexaban** (blue), and optimized structure using Grimme's B97D method (light blue); (**g**) Molecular superimposition of the in solution optimized molecular structure of **fidexaban** Becke3LYP (blue), and Grimme's B97D method (light blue); (**h**) Overall shape of the **darexaban** computed at the Becke3LYP/6-31++G(d,p) level of theory; (**i**) Overall shape of **darexaban** computed at the Grimme's B97D/6-31++G(d,p) level of theory; (**j**) Overall shape of **letaxaban** computed at the Becke3LYP/6-31++G(d,p) level of theory; (**k**) Overall shape of **tanogitran** computed at the Becke3LYP/6-31++G(d,p) level of theory; (**l**) Molecular superimposition of the Becke3LYP optimized molecular structure of **tanogitran** (blue), and optimized structure using Grimme's B97D method (light blue); (**m**) Overall shape of the **SAR 107375** computed at the Becke3LYP/6-31++G(d,p) level of theory.
